# Playing It Differently: Using Performance‐Based Interviewing to Engage Children in Healthcare Research

**DOI:** 10.1111/hex.70922

**Published:** 2026-10-26

**Authors:** Claire Treadgold, Jessica Nikolovski, Valsamma Eapen, Vasiliki Koutoulas, Constance Smith, Sarah Elliott, Lynn McCartney, Barb Vernon, Karlen R. Barr, Dianna Smith‐McCue, Les White

**Affiliations:** ^1^ Behavioural Sciences Unit, Discipline of Paediatrics and Child Health, School of Clinical Medicine UNSW Medicine, UNSW Sydney NSW Australia; ^2^ Starlight Children's Foundation Sydney NSW Australia; ^3^ Sydney Clinical Trials Centre, Faculty of Medicine and Health University of Sydney Camperdown Sydney NSW Australia; ^4^ Susan Wakil School of Nursing and Midwifery, Faculty of Medicine and Health University of Sydney Camperdown Sydney NSW Australia; ^5^ Academic Unit of Infant, Child and Adolescent Psychiatry, South Western Sydney Local Health District Sydney NSW Australia; ^6^ Faculty of Medicine, School of Psychiatry University of New South Wales Sydney NSW Australia; ^7^ Ingham Institute for Applied Medical Research Liverpool NSW Australia; ^8^ The University of Notre Dame, School of Medicine Sydney Sydney NSW Australia; ^9^ Children's Healthcare Australasia Mitchell Australia; ^10^ The Sydney Children's Hospitals Network Sydney NSW Australia; ^11^ Womens & Children's Hospital Adelaide SA Australia; ^12^ Australian Institute of Health Innovation, Macquarie University Sydney NSW Australia

**Keywords:** child‐centred research, co‐design, creative methods, paediatric patient experience, PREMs, qualitative interviewing

## Abstract

**Introduction/Background:**

Capturing children's healthcare experiences remains inherently challenging. Traditional interview approaches are often poorly suited to younger populations and their developmental needs, limiting opportunities for them to meaningfully express their perspectives. This can result in children's voices being underrepresented in research and healthcare. To address this concern, we trialled a novel, child‐centred methodology, using professional performers trained in engaging hospitalised children (‘Captain Starlight’). The approach was implemented within a national paediatric patient‐reported experience measure (pPREM) development project, which served as a case study for the application.

**Methods:**

This study examines a child‐centred data collection methodology and documents the process, benefits and challenges of a creative interviewing approach designed to engage children in qualitative research. The methodology was implemented during a larger project to co‐design a pPREM, which serves as context for this case study. Professional performers (‘Captain Starlights’, herein ‘Captains’), trained in child‐centred communication, conducted semi‐structured interviews while remaining in character as visitors from another planet who are unfamiliar with hospitals. This mythology aimed to position children as the ‘experts,’ encouraging them to explain what matters most during hospital experiences. Interviews incorporated play, storytelling, and age‐appropriate activities to facilitate engagement. To understand the usefulness of this approach, we conducted follow‐up qualitative interviews with Captains and used reflexive thematic analysis to explore how the method shaped interactions and data collection.

**Results:**

The creative interviewing approach enabled flexible, child‐led interactions, with Captains adapting their communication styles to suit the child's age, personality, and context. The use of play and character‐driven narrative helped overcome children's unfamiliarity with providing formal feedback, fostering openness and sustained engagement. However, challenges included balancing character immersion with data collection, navigating consent processes, and maintaining clarity around the interviewer's role.

**Conclusion:**

This novel interviewing methodology demonstrates how playful, imaginative approaches can facilitate meaningful engagement with children in healthcare research, enabling richer insights into their experiences. The approach created a safe and empowering environment in which children could articulate their needs and preferences. It forms part of a broader multi‐stage project to co‐develop a paediatric PREM, with this context allowing the methodology to be successfully piloted as a transferable approach for engaging children in healthcare research.

**Patient/Public Contribution:**

While this methodological study did not directly involve patients in its evaluation, it is embedded within a larger co‐design project centred on capturing children's voices to develop a paediatric patient‐reported experience measure (pPREM). JN served as the patient and community representative for the pPREM project and this study, contributing a lived‐experience lens to the research and providing qualitative methodological oversight to support patient‐centred study design, analysis, and interpretation.

## 1 Introduction

It is widely recognised that children comprise a significant group of patients within the healthcare system; however, their needs are largely different to those of adult patients [1, 2, 3]. The opinions of paediatric patients are often underrepresented in healthcare as they are less frequently asked about their care experiences compared to their adult counterparts [1, 4, 5]. There is a commonly held assumption, whether justified or not, that paediatric patients lack the vocabulary, capacity, and formed opinions to answer questions meaningfully [5, 6, 7].

The Children and Young People's Rights in Healthcare Services Charter in Australia and similar initiatives across the globe highlight that children experience illness, injury and disability in a way that is different to adult patients [8]. The charter recognises that every child has the right to their best interest being considered, an avenue to express their views, to be involved in decision‐making, and to participate in education and recreation as would a child who is not in hospital [8]. As it stands currently, there is a limited availability of tools that allow children to voice their experiences of hospital in a way that is age‐appropriate and does not rely on a parent or caregiver answering the survey [9, 10, 11]. To create a methodology that properly captures and evaluates the experience children have as a hospital patient, the children themselves would need to be at the forefront of the design and implementation of the new methodology or dedicated tool to ensure it is developmentally appropriate for their future use [12].

One way of engaging children that allows them to voice their opinions about healthcare in an age‐appropriate manner is the use of performance, play therapy or similar creative activities. There have been studies that explore utilising performers, musicians and play activities when engaging children in hospital [13, 14, 15, 16]. Findings from these studies have supported using creative and play‐based activities as a more developmentally appropriate way of engaging a young cohort [13, 14, 15]. Additionally, it has been demonstrated that facilitators to communicating with paediatric patients include ensuring trust, respect, and shared decision‐making [171]. In recognition of this, we proposed that using performers as interviewers of hospitalised children and leveraging their skills in child‐centred engagement with an emphasis on play would result in a positive and productive method for qualitative data collection. The combination of performance skills, dedicated training and an emphasis on child‐led interactions would overcome many of the traditional barriers to successfully capturing the voices of children in healthcare research.

Starlight Children's Foundation (Starlight) is a not‐for‐profit organisation, with a permanent presence in all Australian tertiary paediatric hospitals. Starlight team members provide positive distraction and social connection for sick children and young people (CYP), aiming to improve their healthcare experience [18]. Captain Starlights (‘Captains’ here) are professional performers who are highly skilled in methods used to interact with children through creativity and imaginative play [19]. Previous research has demonstrated that the mythology of Captains and the emphasis on play and fun create a sense of escapism within the hospital environment for children, while supporting healthcare participation and agency [18, 19, 20]. It highlighted the ability of the Captains to engage with children of varying ages, developmental stages and abilities, as well as their skills in overcoming language barriers and addressing cultural considerations [20, 21, 22].

This paper specifically focuses on the novel methodological contribution of using trained performers (‘Captains’) as interviewers, examining how this creative, character‐based approach shapes engagement, data collection, and the inclusion of children's voices in healthcare research. Although this methodology was trialled within a paediatric Patient Reported Experience Measure (pPREM) development project, the focus of this paper is the interviewing approach itself, with the pPREM serving as the case study through which the methodology was implemented and evaluated.

## 2 Methods

### 2.1 Case Study Project Context and Background

This approach represents a deliberate methodological innovation, shifting from traditional researcher‐led interviews to a performer‐led, child‐centred model designed to reduce power imbalances and support more authentic child participation. It utilises the development of a paediatric Patient Reported Experience Measure as the vehicle to pilot the approach (with details below providing context for the environment in which the methodological approach was applied).

Patient‐reported experience measures (PREMs) are questionnaires that capture how a patient perceives their hospital stay [6, 23, 24]. PREM data can be used at a service and system level to identify models of care that enhance patients' care experience and quality [4, 6, 25]. PREMs additionally have broader implications in policy development, quality assurance and benchmarking hospitals worldwide [23, 26]. However, there is limited use of PREMs in a paediatric setting internationally (‘paediatric PREM’ or ‘pPREM’) [23, 27]. Many pPREMs fail to include a paediatric voice in their creation, thus poorly reflecting the priorities and preferences of young patients [1, 28]. To understand what matters most to children in the Australian context, there was recognition of the need for a suitable pPREM which is not only designed for use in children but also involves them in its development [2, 3]. The development of a pPREM co‐designed with children will effectively provide a voice to paediatric patients, thus addressing their international right to be involved in their own healthcare and improving the experience of CYP in Australian hospitals [29]. The broader ‘Understanding what matters to children and young people when visiting hospital’ study was created through a partnership of representatives from Children's Healthcare Australasia, the Sydney Partnership for Health Education Research and Enterprise (SPHERE) at UNSW, and the Starlight Children's Foundation, as well as consumer representatives, academics, and clinicians. Papers detailing the perspectives of what matters to CYP from Stage 1, and the protocol of the broader project, has already been published [29, 30].

This article focuses on the novel approach adopted in the first stage of the pPREMs project; the methods used to identify what matters most to children when in hospital. Ethics approval was provided by the South West Sydney Local Health District Human Research Ethics Committee (HREC) (2024/ETH00904).

### 2.2 Study Process

Following the completion of an initial literature review, the advisory group discussed the most suitable methodology for engaging with children. It was recognised, from both the literature and practical research experience, that traditional methods of data collection, such as focus groups or interviews, would not be appropriate for children. Instead, a novel research methodology was proposed, utilising the specific skills and expertise of Starlight team members. Starlight Captains are specifically provided with the skills and training to engage with sick children [19, 31]. Their unique situation of being embedded in hospitals provides access to, and familiarity with, children and families. In their role at Starlight, they are accustomed to receiving requests for support with activities outside of their day‐to‐day role (e.g., helping facilitate forums or take part in fundraising events) while always maintaining their character. They are also familiar with research and evaluation, participating in regular internal evaluations and partnering with universities and other research institutions on a range of studies. The longevity of the Captains programme in hospitals‐ spanning multiple decades ‐ and the formal contingency worker status of its team members (including acknowledgement of their child protection/safeguarding training and achievement of any site‐required certification) fosters familiarity and trust with hospital staff, enabling safe access to and interaction with patients. This combination of skills, training, profile and exposure to research presented a clear opportunity to utilise Captains in research regarding paediatric patient experiences.

This paper will detail the novel research methodology utilised by Captains in their interactions with children throughout the pPREM study [29, 30]. It aims to document and demonstrate the value of using creative, child‐led methods to ensure that the voices of children are captured in research and translated into clinical practice. We will share the different tailored approaches used and provide critical feedback and insights from the Captains who participated.

As previously outlined, Captains are employed by Starlight for their skills in engaging with children and adolescents in healthcare settings and are provided with extensive training to support these interactions [14, 19, 20, 21, 22]. Additionally, there are principles that also had to be adhered to as part of their employment role and were therefore brought into the research. For example, all Captains are known as Captain Starlight, and wear the same, easily identifiable superhero‐style uniform. This means to children they are ‘interchangeable’, but consistent. They cannot break character and are instructed to lean into the mythology of being from ‘Planet Starlight’ in all interactions.

During Stage One of the study, a protocol was drafted and shared with involved hospitals (Appendix A). The National Research and Evaluation Manager at Starlight (CT), who has extensive experience of the Captain programme and strong internal relations with the Programme Manager at each hospital, briefed them about the project and supported them to recruit a small group of interested Captains from their teams at each location. It was entirely optional for the Captains to participate. All activities were conducted in their regular employment hours. They were encouraged to address any questions or concerns to both the Programme Manager and/or the National Research and Evaluation Manager. In addition, the site Principal Investigators (PIs) were available to support recruitment and consenting processes (allowing the Captains to remain ‘in character’).

Working with other Starlight team members aligned to the Captains programme, a series of materials was produced to support them on this project. Materials included a video introduction and overview of the project, an online video briefing meeting, a simplified version of the protocol and a ‘frequently asked questions’ sheet (Appendix B). As per the HREC approval, a Starlight hospital‐based Programme Manager was present at each site to support the PIs with recruitment and consent/assent, and the Captains throughout the interview process.

The Captains were given a briefing to allow the interaction to be ‘child‐led’ utilising play, art, craft and other age‐appropriate tools. The Captains followed the well‐developed Starlight ‘mythology’ whereby the children are made to believe that Captains are not from Earth, but rather are visitors unfamiliar with their surroundings (‘landing their rocket ship on the roof of the hospital’) [19]. Keeping with this mythology allowed the child to become the ‘hospital expert’ while the Captains framed their questions as a reflection of their supposed naivety. Interviews followed a semi‐structured guide that was pre‐developed by the advisory group whereby children were asked to imagine the best/worst hospital experience, with relevant follow‐up questions (Appendix B). Captains are accustomed to adapting their interactions to different needs of children and were encouraged to use those skills. [19] The activities were tailored to be inclusive of children with disabilities where required.

During Stage One, when the advisory group discussed the methodology and the use of the Starlight mythology, it was proposed to develop a video for the Captains to use to engage the children as a mechanism to frame the conversation (Appendix C). The video was a 1‐min animation, again leaning into the mythology, with ‘Admiral Starlight’ speaking directly to the child, explaining that they were being asked to take on a ‘special mission’ and report back to the Captains about what hospitals on Earth are like. It was posed as a fun challenge, giving the child the role of the expert.

The interactions with children were always undertaken with two Captains present in accordance with Starlight's Child Safeguarding principles (Appendix D). Each interaction was audio recorded (the decision was deliberately made not to video children as this could be intimidating or distracting). The interviews took approximately 15 min to complete; there were no follow‐up interviews. Depending on the preference of the child and/or the parent and guardian, parents and guardians could be present during the interview. The results of the interviews themselves are published elsewhere [29, 30].

### 2.3 Check‐In Interviews

While piloting this novel approach to gathering data in young populations, it was considered important by the broader advisory group to document the methodology and review it for future use. A recommendation was made to review the role of the Captains in the project and document and analyse the success of their approach in engaging children. As a member of the pPREMs project Advisory group, and already having a professional working relationship with Captains, the Starlight National Research and Evaluation Manager (CT) volunteered to manage this process. At a mid‐way point through the broader pPREM Stage 1 project, a ‘check‐in’ was held by CT with a randomised sample group of five Captains. Using a semi‐structured format, the intent was to assess how the Captains felt the project was progressing and to identify any issues or concerns with the methodology. Questions focused on how the children were responding to the approach, the types of activities they had chosen to do, what the depth of their responses were, any challenges in understanding and the Captains own reflections on the approach and its success in engaging children. A specific consultation was also held with each Hospital Programme Manager (who were in regular contact with the PIs about the project) in addition to ongoing regular communications. A summary of this combined information was shared with the advisory group. As a result of feedback from these check‐ins, it was noted that some of the Captains had spoken about how one of the interview questions (about how children liked to give feedback) was ‘awkward’ to deliver in a child‐friendly way. In response, the advisory group developed a training unit on ‘asking questions’ which was recorded and shared to assist the participating Captains, with the support of the Starlight National Research and Evaluation Manager.

### 2.4 Data Collection

Following the completion of the Captains' interviews with children for the pPREMs study (Stage 1), all Captains involved in the project were invited to participate in an interview, reflecting on their experience. This process is in line with standard Starlight procedure, with debriefing and reflection activities (with both internal and external facilitators) are a feature of the Captain's employment. The invite was optional, and of the 14 Captains who participated in the study nationally 5 agreed to be interviewed. The interviews were conducted over online video by a member of the Starlight research team and consent for the interviews to be recorded and transcribed was provided. Each interview was approximately 30 min. All answers were anonymised.

### 2.5 Data Analysis

Data from the final interviews with Captains were analysed using inductive, reflexive thematic analysis following Braun and Clarke's approach, which supports an iterative process of familiarisation, inductive coding, and theme development. The core team repeatedly read transcripts to ensure immersion in the data (JN, CT, CS, VS). Data segments were then coded inductively by JN, CS, and VK, who undertook initial coding independently before meeting with CT to compare interpretations and consolidate codes.

The analysis was led by JN, a consumer researcher with a strong background in youth advocacy and extensive experience engaging children and young people in health research and policy. This positioning was critical to ensuring the methodology remained grounded in principles of participation, accessibility, and respect for children's voices. JN's expertise supported the design and interpretation of the approach, prioritising the authenticity and relevance of children's contributions. CT, a Research Manager with operational expertise in delivering child‐focused programmes within hospital settings, helped JN interpret findings. CT provided important contextual insights into the practical realities of implementation, the dynamics of child–interviewer interactions, and the broader care environment, supporting interpretation of the data beyond the immediate research context.

Emerging findings and interpretations were subsequently brought to the wider multidisciplinary project team, including researchers, clinicians, and consumer representatives, for discussion and refinement. This iterative process ensured that conclusions were rigorously interrogated, contextually grounded, and aligned with the overarching aim of informing the development of a robust and meaningful pPREM.

## 3 Findings

The five Captains who agreed to be interviewed were all experienced in the Starlight position, with a minimum of 4 years ranging to 16 years in the role at the time of the interview. They were located in four different hospitals and conducted an average of 6 interviews each, with children aged between 6 and 13 years old. Across their own interviews on the project, Captains reflected on both the strengths and challenges of adopting a character‐based, child‐led approach to qualitative interviewing. This included addressing issues related to engagement, communication, role boundaries, and practical implementation within the hospital context. The themes presented below highlight how this novel interviewing method influenced both engagement and the quality and nature of the data collected.

### 3.1 Balancing Imagination and Inquiry: How Character‐Based Interviewing Shaped Engagement and Data

Captains enjoyed portraying mythological characters to engage children. However, many Captains recognised there was an intricacy in how far to take their character, whilst remaining on topic to address the goal of the research. They admitted that there were limits to their usual silliness and fun. Being overly silly could distract some children, while taking a serious approach might make others feel intimidated or like they were in trouble:

If you went too silly, kids wouldn't focus.

(Captain A)

One Captain recognised that for this kind of qualitative interviewing, keeping it really light, so not asking super direct questions with the kid works really well.

(Captain B)

Captains mentioned that the children's existing knowledge of Starlight, given their ongoing presence throughout the hospital outside of the study, helped build rapport. They observed that this familiarity was beneficial, enabling more open conversations with the children since they already trusted the Captains.

We've met them all before.

(Captain C)

All of the patients we talked to had already met Captain Starlight…you didn't have to build rapport.

(Captain D)

While it was a bit of a learning curve to interview as a Captain, I think it made the child naturally more open and receptive to us and naturally trusted us a bit more.

(Captain B)

Additionally, using Captains as interviewers was beneficial as they are trained to confidently interpret patients' body language and respond to such cues. Captains recognised *‘lots of fiddling’ (Captain B)* as typical child behaviour, and thus were not put off by this during the interviews. Children were reported to be *‘chatty and open’* and *‘maintained eye contact really well…engaged with the conversation’ (Captain B)*. Captains felt capable of noticing changes in physical behaviours that would indicate a need to stop the interview, and they checked in with their participants throughout the process.

I feel like we are adept enough to be able to see [discomfort]…we always would mention that the interview could be stopped at any time.

(Captain E)

Captains are pretty good at just engaging that emotional intelligence.

(Captain A)

Additionally, Captains felt they were receptive to different children's personalities, and this was perceived to be their interviewing strength:

Some kids were quieter than others…in terms of both physical loudness and the willingness to speak.

(Captain E)

To stimulate conversation and help participants feel more comfortable, Captains implemented games and activities. Some interviews were not sedentary as children wished to move around whilst speaking to Captains. Some children could not leave their bed, so Captains brought games to them.

Examples of games included:

I would ask the question, and for every answer that we got, she would be able to tip a bucket over the other Captain's head.

(Captain D)

Another Captain remembered asking a child to *‘build something out of Lego’* which was *‘engaging for [the children] and they didn't feel bored throughout it…I feel like it helped their answering a lot’ (Captain B)*.

### 3.2 The Value of Incorporating Animation to Explain Research to Children

Captains were relieved when they received the instructional video for the study (as described in 2.2 and Appendix C). It allowed them to draw upon their imaginative spirit to conduct interviews, whilst maintaining their usual boundaries with children.

Once we saw that video (with Admiral Starlight) and saw the questions… we realised that this is kind of a bit more imagining, rather than actual experience. That felt quite reassuring that we could still be very much Captain Starlight about it. And have that sort of layers of protection and kind of distance.

(Captain E)

Children also enjoyed seeing a cartoon of Admiral Starlight who informed them of the study.

We had a video that we showed that had been created…the cartoon with Admiral Starlight…kids liked that.

(Captain A)

### 3.3 One Child at a Time: Benefits of One‐On‐One Interviewing

Early in the development of the larger project, it was decided that children would be interviewed individually rather than in larger focus groups. The Captains endorsed this decision as there *‘was a concern [children] would influence each other's answers’ (Captain D)*.

Within the one‐on‐one interviews, due to child protection rules (outlined in Appendix D), Captains worked in pairs. Some Captains agreed that this was a strength of the methodology as they saw it as an opportunity to bounce off one another and develop an engaging environment. Others reported that two Captains overwhelmed some children, thus found it more beneficial to have one Captain take the lead whilst the other acted as an observer.

[I preferred] one‐on‐one because it seemed easier for the kid because I think it was a bit overwhelming having two people sitting there, both interviewing them.

(Captain A)

### 3.4 Recruitment Processes

Recruitment processes were identified as a challenge by multiple Captains. Some Captains reflected that it was occasionally difficult for the PIs or Programme Managers to both inform participants and obtain subsequent consent for an interview on the same day. The logistics of obtaining consent if the parent was not there, or asleep, was also challenging. Additionally, juggling interview timings with hospital school or other procedures and before the patient was discharged acted as a barrier to obtaining consent and scheduling interviews.

It was definitely [difficult] finding parents to get the sign off to do it. I mean that was the biggest barrier.)

(Captain B

At some hospital sites, there was confusion about whose role it was to identify and recruit participants.

Whose job is it to identify the families? Because that was really confusing.

(Captain E)

Hospital staff member (recruiting)… wasn't really working.

(Captain E)

One complication was as that Captains are to ‘remain in character’ at all times, they could identify participants, but were reliant on the PIs or Programme Managers to coordinate the formal consent activities (they did seek verbal assent from the children at the beginning of each interaction).

Conversely though, some Captains appreciated that recruitment and consent processes were conducted by other hospital staff as it allowed them to maintain their fun character:

Consent was being taken by people who weren't Captains… that made it very easy to just come in and be like, we're the fun.

(Captain D)

Keeping in character did create uncertainty amongst some Captains about how to capture the child's perspective without delving into topics outside the scope of their usual playful role.

We never break character, and we never ask medical questions… [the interview] was putting us into a very different relationship space with these kids… we're asking them to do something that we wouldn't ask normally.

(Captain A)

### 3.5 Looking Ahead: Suggestions to Modify Processes for Future Research

Captains suggested that a useful and creative way for children to understand and answer questions in the future would be to use more visual aids, such as whiteboards. Furthermore, Captains brainstormed ways to recognise participants for their time, such as a *‘certificate’ or ‘sticker’*, after reasoning that a prize draw may seem like *‘bribing’* or *‘giving kids something for their answers’ (Captain C)*.

One Captain believed that future research should include siblings' perspectives.

It would have been very interesting… what the siblings think of the hospital experiences as well.

(Captain D)

Finally, though Captains recognised the project as crucial to capturing the often‐overlooked voice of children, there was uncertainty amongst some about the purpose of the interviews and who was conducting the research. Some felt they did not have enough information about the project to answer questions from participants and their families:

From the trickled down information we got… I personally feel fortunate that no one asked us questions about the process or the interview at the time.

(Captain C)

They also asked to be briefed alongside ward staff, so hospital staff understood that Captains were conducting research in addition to their usual tasks in hospital. Captains shared that a more detailed briefing of the study could have *‘helped (them) in structuring interviews more… [and] our planning’ (Captain C)*.

Overall, as the Captain's confidence grew, and recruitment hurdles were overcome, they felt it was a positive experience that they would like to participate in again.

I really enjoyed the process. I would love to get involved again…. great idea to get these kids' opinions and hopefully, you know, make change if change needs to happen.

(Captain E)

I think it's so important…it's really easy for the children to get overlooked in the hospital… the kids really see and hear and feel so much more than we realise, a lot younger than we realise, too…if it happens again, I would love to be involved.

(Captain C)

Once we got the ball rolling, the ball was rolling! It was great.

(Captain D)

## 4 Discussion

This study demonstrates how a creative interviewing approach can be used to engage children in healthcare research. By leveraging the child‐centred, playful and imaginative communication style of the Captains, the methodology aimed to facilitate an environment whereby children felt comfortable expressing their needs, concerns and wishes during their hospital experience [14].

### 4.1 Collaborating with Non‐Researchers

Incorporating professional performers like Starlight Captains into a qualitative research framework was an innovative approach that brought both advantages and challenges. The concept of Captains is familiar to many hospitalised Australian children and even though the individual performers differ, the children recognise the character (not the person). This enabled flexibility for different Captains to participate in the research. Additionally, Starlight Captains pre‐existing relationships with many of the participants eliminated the need for extensive rapport‐building, allowing interviews to proceed more smoothly. Children viewed Captains as friendly and trustworthy figures, which likely contributed to more open and honest responses. They were familiar with playing with them and therefore happy to ‘follow’ the activity and answer questions openly.

However, this familiarity also raised concerns about bias. The children's positive associations with Captain Starlight may have influenced their responses, introducing a potential bias in the data. The Captains (seen as figures of fun and support) may have inadvertently shaped the children's responses, encouraging them to present a more positive outlook on their hospital experiences.

Furthermore, the dual role of the Captains (as both entertainers and researchers) led to some confusion about their responsibilities. Several Captains expressed initial uncertainty regarding maintaining their Captain personas and mythology while asking research‐focused questions, as this was outside the scope of their usual work. This highlights the need for more comprehensive training and briefing for the interviewers in future, as well as ongoing supervision and support by the primary research team, ensuring they are confident to handle both roles effectively.

### 4.2 The Role of Play

One of the key innovations of this project was the use of play as a form of connection and communication during interviews. Research suggests younger children who may struggle to articulate complex thoughts in a traditional interview setting responded positively to the integration of play as a communication tool [7]. Similarly, Captains reported that games, drawing, and imaginative scenarios provided a familiar and non‐threatening medium through which children could express themselves. This proved particularly beneficial for those children who might have been otherwise overwhelmed by the clinical environment or intimidated by unfamiliar researchers. The implementation of Captain Starlight as a data‐collection vehicle was reported to achieve the aim of engaging the children.

### 4.3 Child‐Centred and Age‐Appropriate Activities

The methods used in the study highlighted the value of the Captains being able to tailor communication styles to different age groups. The beauty of using creative activities rather than a standard survey, for example, is that they can be tailored to age, development and education level. For younger children, creative activities such as drawing or games proved effective in keeping the children engaged and facilitating open‐ended responses. Being able to choose the right activity for each child reiterated the child‐centred nature and expertise involved in the Captains approach. The use of the ‘Admiral Starlight’ video was seen as beneficial, with strong feedback on how it drew in and engaged children in the task (Appendix C).

### 4.4 Benefits and Challenges of Individual Interviews

While there had initially been plans to conduct the interviews in groups, the practicalities of coordinating this with minimal impact on the children (e.g. around tests, procedures and rest periods) worked against this. However, ultimately it appears that having to perform interviews one‐on‐one may have been beneficial. It overcame concerns that a group setting may have influenced individual responses, with children potentially mimicking the answers of their peers. The individual interviews allowed for more personalised interactions, with Captains able to adapt their approach based on the specific needs and personality of each child. This flexibility was crucial in ensuring that each participant felt comfortable and understood throughout the interview process.

However, the individual interview format also presented logistical challenges, particularly in obtaining consent from parents or guardians to conduct the interview when they were available. While gaining consent is a well‐known challenge in research, this was exacerbated by the Captains having to stay ‘in character’ and rely on support of additional research team members. Short‐term hospital stays further complicated recruitment, with many eligible participants leaving the hospital before interviews could be conducted. These barriers highlight the need for more streamlined recruitment processes and an impartial member of the team who could gain consent in future studies.

### 4.5 Value of Using Performers to Facilitate Interviews

The findings from this study offer valuable insights for future research involving children. It is clear that the novel utilisation of Starlight Captains in research is useful in building rapport and engaging young participants. The use of creatively trained individuals, who the children were familiar with, allowed for active engagement and rapport building. With the children feeling relaxed and at ease in the Captains presence, they were able to be open and expressive about their true hospital experience. However, there are areas for improvement. For example, the Captains suggested incorporating additional creative methods for children to express their feedback, such as using visual aids or alternative activities. These methods could help bridge the gap between abstract concepts like ‘feedback’ and tangible, age‐appropriate ways for children to share their thoughts. While rapport and familiarity were strengths, they may also have introduced social desirability bias, with children potentially moderating their responses due to their positive perceptions of the Captains.

Additionally, the study highlighted the importance of training and debriefing for Captains involved in future research. Despite efforts to provide introductory and training materials, some Captains felt they did not have enough information about the study's objectives or how to conduct interviews effectively, which led to some uncertainty during their initial interviews. Future studies should include more detailed briefings and further opportunities for Captains to ask questions and receive feedback on their performance. Consideration could be given to selecting and developing a pool of Captains specifically for supporting research activities (including ensuring they are part of planning and design phases). This would not only improve the quality of the data collected but also ensure that the Captains feel more confident and supported in their dual roles as entertainers and researchers.

The use of use performers and/or play therapy to engage children in healthcare research has been evaluated in several other studies [13, 14, 15, 16]. Findings from such studies have favoured the use of creative and play‐based activities over traditional survey methods when engaging children in research. The methodology outlined in this paper differs from others in the literature as it is the first of its kind to utilise performers who have inherent training and familiarity in engaging children in a hospital‐specific setting. We know that children's voices in hospital have been historically difficult to assess. Thus, this unique utilisation of Starlight Captains to assess children's experience of hospital is novel and valuable.

### 4.6 Strengths and Limitations of This Study

The positivity bias toward the Starlight Captains is acknowledged as a potential limitation of the study. To address this, future studies might consider using similar performers or facilitators whom the children do not associate with a specific organisation, to reduce the risk of bias while maintaining the playful and relaxed environment that proved effective in this study.

Additionally, interviewing the researchers involved in this study would have provided an alternate and broader perspective and hence strengthened the evaluation of this novel methodology. Their reflections would have provided insight into how the approach performed, strengths, challenges and the quality of data generated. Experienced researchers would be well positioned to provide insight into the applicability of this approach in other health services and in future child‐friendly research generally.

### 4.7 Future Direction

Our findings align with a growing body of research demonstrating that the manner in which patient‐reported data are collected can substantially influence participation, engagement and accessibility, particularly for children, reinforcing the need to move beyond traditional question‐and‐answer approaches when seeking to understand young people's healthcare experiences [32].

While no language barriers were reported in this study, there is potential to explore further how this approach could be used to help incorporate culturally and linguistically diverse voices, which is a recognised gap in health research and clinical settings [33]. Research into the Captains working with Indigenous children suggests that there is potential benefit for their performance skills, which are also often relied on for non‐verbal children [33]. This would be an interesting area to explore further.

Additionally, it is recognised that the Captains are limited both in number and geography. In Australia, they currently only have a permanent presence in tertiary paediatric hospitals, creating questions around the viability and scalability of using Captains in this kind of child‐centred healthcare research methodology. While technology may have a role to play in access, there are undoubtedly benefits with face‐to‐face interaction. There is value then in exploring the possibility of identifying the elements of their training, skills and principles in engaging with children, which could be used by other individuals or organisations to apply in a similar manner.

## 5 Conclusion

This study describes and reflects on the value of a novel, creative methodology for engaging children in healthcare research. While originally developed within a national pPREM co‐design project, the use of trained performers in character‐based interviewing offers a transferable approach to capturing children's voices in ways that are developmentally appropriate, engaging, and responsive. Whilst challenges were acknowledged, and a number of recommendations for future research were suggested, numerous positive outcomes were identified in the methodology that helped to address the challenges of engaging children in hospital. There is potential to further explore how imaginative play and creative approaches can assist in capturing the voices of children to enhance meaningful child participation in healthcare research.

## Author Contributions

**Claire Treadgold:** conceptualisation, writing – original draft, writing – review and editing, validation, methodology, project administration, resources, supervision, investigation, formal analysis, data curation. **Jessica Nikolovski:** writing – original draft, writing – review and editing, validation; methodology, formal analysis, project administration, supervision, data curation. **Valsamma Eapen:** methodology, writing – review and editing, funding acquisition, project administration. **Vasiliki Koutoulas:** formal analysis, writing – review and editing, writing – original draft, validation. **Constance Smith:** writing – original draft, writing – review and editing, formal analysis, validation. **Sarah Elliott:** writing – review and editing, project administration. **Lynn McCartney:** writing – review and editing, investigation, project administration. **Barb Vernon:** writing – review and editing, funding acquisition, project administration. **Karlen R. Barr:** writing – review and editing, project administration. **Dianna Smith‐McCue:** writing – review and editing, investigation, resources, project administration. **Les White:** writing – review and editing, project administration, funding acquisition, methodology, supervision.

## Ethics Statement

Ethical approval obtained from the South Western Sydney Local Health District Human Research Ethics Committee (SWSLHD HREA) (2021/ETH10836) and local research governance approval was obtained for each participating hospital site. All data collected was de‐identified to ensure confidentiality.

## Conflicts of Interest

The authors declare no conflict of interest. Dr Claire Treadgold is employed by the Starlight Children's Foundation.

## Supporting information


Supporting File 1

Supporting File 2

Supporting File 3

Supporting File 4

## Data Availability

Data are not publicly available as ethics approval was not granted for data sharing.
