# Supplementary material for: Playing It Differently: Using Performance‐Based Interviewing to Engage Children in Healthcare Research
Source: Health Expect. 2026 Oct 26;29(5):e70922. doi: 10.1111/hex.70922 (PMC13700104; doi:10.1111/hex.70922)
Supplement: Supplementary file 1 [file HEX-29-e70922-s001.pdf]

Starlight & CHA PREMS Research Project

Briefing notes for Programs team

Background

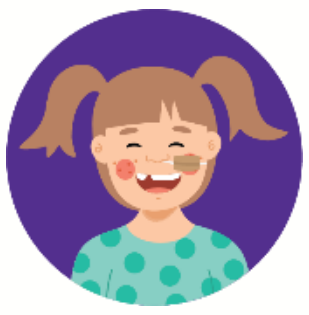

It is important to listen to what children and young people have to say about their hospital experiences. However, most hospitals capture feedback from parents rather than from the child or young person. This is because the feedback questions have been developed only for use by adults.

Starlight have partnered with the Children's Healthcare Australia (CHA) and Sydney Partnership for Health Education Research and Enterprise (SPHERE) to develop a set of feedback questions that can be used by children and young people.

What is the research project about?

We will develop a set of feedback questions (which are called a 'Patient Reported Experience Measure' or PREM) to be used by children and young people across Australia. This PREM will capture the experiences of children and young people when they come to hospital. And in turn that info will be used to help improve the experience for all children and young people.

To develop the questions in our PREM, we first need to understand what matters to children and young people when visiting hospital. We will do this by Captain Starlights & Livewire Facilitators talking to children and young people. We will use a short video as a conversation starter and then an activity (child-led) to guide the conversation.

Which children are participating?

Only children whose parents have given consent (including completing ethics forms) can participate. We need about 15 children at each site. The relevant Hospital Program Managers and hospital team member who is acting as the "Principal Investigator" will be responsible for ensuring that the right forms are completed. Then the HPM will let the Captains/Facilitators know which child/children to engage with. It can be in the SER or bedside. The Captains (as always) stay completely in character throughout all the interactions.

The **Hospital Program Manager** will be responsible for:

- Ensuring only children who have permission participate,
- Observing where appropriate to ensure to all is going smoothly
- Answering any questions from parents
- Identifying if any child or young person becomes distressed during the activity and inform the parent immediately
- Saving and sending through the audio recordings

How will the activity/conversation work?

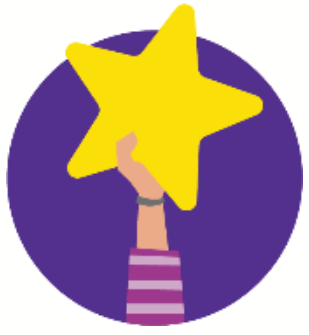

Participating children will complete an activity, either in the SER (could be as an individual or in a small group) or individually bedside in more of a Rocket Round format.

We are really using the mythology and asking children to explain to Captain Starlight what being in hospital on earth is like. The interaction will start with a video on the tablet from "Admiral Starlight" explaining that we are asking the child to take part in a mission to tell us more about hospitals. It is then up to the Captains to run with whatever child-led activity they think will work best to facilitate the conversation about what they think is good/bad/etc.

The activity is up to you and might be different each time. It could be a drawing, craft, Lego, puppet or even a mock TV interview. It is up to the Captains to use their experience and choose what will work best in that situation, with that child. There is a list of questions below that the Captains should cover as part of the activity – please note, they do not need to be word for word, it is more about the concept!

The session will need to be audio recorded – this can be done using the voice memo app on the tablet. Don't forget to save it when finished.

What about older children/teenagers?

Livewire Facilitators will host casual hang out chats with teens (individually or in a group) to capture this information, rather than Captain Starlight running a creative activity. Again, it could be a conversation with an individual, or done with a small group.

Depending on numbers, we will also run some chats on Livewire Online for teens who are unable to attend in person.

What questions will Captain Starlight/LW Facilitators be asking?

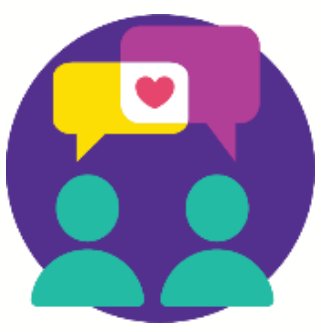

These are just a guide of the topics we would like to capture – but please make it work for you and the child! It doesn't have to be word for word or in this order. Choose words and phrases the child will understand or suit the game you are playing. The flow of the activity and conversation should be organic and child led where possible.

**Main concept to cover**		**Suggested questions to cover**
**1**	**Imagine the BEST hospital experience that**	What is like when you arrive at the hospital?
		What does it look like?

Starlight Children's Foundation

	**you can – a perfect hospital …**	What are the doctors/nurses like?
		How do people talk to you/ speak with you?
		How involved were you in discussions that affect you?
		How involved were you parents and carers in decisions?
		Were your parents involved as much as you wanted?
		Were your parents involved as much as you wanted?
		Why is that good, or important?
2	**Imagine the WORST hospital experience that you can …**	What is like when you arrive at the hospital?
		What are the doctors/nurses like?
		What is that environment like?
		How do people talk to you/ speak with you?
		How involved were you in decisions that effect you?
		Why is that bad, / how does that make you feel when that happens?
3	**Summarise the key things discussed by the child and ask them which are the most important**	Has anything has been missed that is important?
		Of all the things mentioned, which are the most important to you that hospitals get right?
4	**Ask how they like to provide feedback or tell people what they think? What's the best way to share?**	Do you want to tell the hospital about (or provide feedback) about your experience when you visit?
		How would you like to tell them? What's the best way you can think of sharing your thoughts?

What if they go into detail re their treatment or get upset?

Just like any interaction with a child, you know how to guide them back into a neutral space and focus on the fun. Lean into that mythology and get them to tell you more about other things related to their hospital experience.

If they get upset, please stop and let the HPM know. They have support options ready for the child and family.

If you feel concerned about anything they share, as always, please debrief with your HPM or tap into EAP.

What will the interviews be used for?

We are going to collect all the information from the six sites participating around Australia and work out from it what is most important about a hospital experience to children and young people. This will then be used to create a PREM containing questions that kids want to answer, delivered in a way they want to participate.

Ultimately, this will mean national data that can be used to help improve the hospital experience for all children and young people.

Questions?

No question too big or small! We have some supporting videos to watch or check with your HPM .

You can also contact Claire Treadgold in R&E –

Email: claire.treadgold@startlight.org.au

Phone: 0417204476

Starlight Children's Foundation
