# Supplementary material for: Playing It Differently: Using Performance‐Based Interviewing to Engage Children in Healthcare Research
Source: Health Expect. 2026 Oct 26;29(5):e70922. doi: 10.1111/hex.70922 (PMC13700104; doi:10.1111/hex.70922)
Supplement: Supplementary file 2 [file HEX-29-e70922-s002.pdf]

Understanding what matters to children and young people when visiting hospital

Development of a National Paediatric Patient Reported Experience Measure

Collaborative research group:
Sydney Partnership for Health, Education, Research and Enterprise (SPHERE)
Starlight Children's Foundation
Children's Healthcare Australasia (CHA)

Project Sponsor: Children's Healthcare Australasia (CHA)

Children's Healthcare Australasia is a not-for-profit organisation whose members include approximately 98 paediatric services across Australia, including all of the Children's Hospitals as well as paediatric units within General Hospitals, in metropolitan, regional and rural areas. Each year, CHA members provide care for more than 60% of total Australian public paediatric inpatients between the ages of 0-16 years. Our members provide a comprehensive range of healthcare services including inpatient care, emergency care, outpatient specialty care, community child health services and child & adolescent mental health services.

Address: Unit 9/25-35 Buckland St,
Mitchell, ACT 2911, Australia
Contact: 02 6185 0325

PROJECT TEAM

Coordinating Principal Investigator:
Name: Professor Valsamma Eapen
Position: Chair, Infant Child and Adolescent Psychiatry, University of New South Wales
Head, Academic Unit of Child Psychiatry, South West Sydney (AUCS)
Stream Director, Early Life Determinants of Health, Sydney Partnership for Health, Education, Research and Enterprise (SPHERE)
Director, Program 1, Co-operative Research Centre for Autism (CRC)
Director, BestSTART-SW (Systems Transformation and Research Translation - South West Sydney) Academic Unit
Institution: University of New South Wales, Infant Child and Adolescent Psychiatry, 30 Botany Street, Randwick, NSW 2031

Lead Investigator / Researcher:
Name: Sarah Elliott
Position: Project Coordinator
Institution: Children's Healthcare Australasia

Principal Investigator:
Name: Dr Eden Robertson
Position: Postdoctoral research fellow / 2020 Fulbright Future Scholar Research & Evaluations Manager, Starlight Children's Foundation
Institution School of Women's and Children's Health | Faculty of Medicine, University of New South Wales;

Principal Investigator:
Name: Dr Ann Dadich
Position: Associate Professor, School of Business
Institution: University of NSW

TABLE OF CONTENTS

Table of contents 2
1. Summary 2
2. Background and rationale 3
3. Study aims/objectives 4
4. Participating sites 4
5. Study design 4
5.1 Study type 4
5.2 Expected study duration 4
5.3 Data source and population 4
5.4 Recruitment and screening 5
5.5 Inclusion criteria 5
5.6 Exclusion criteria 5
5.7 Consent process 6
6. Study procedures 6
6.1 Steering committee 6
6.2 Focus group/interview composition 6
6.3 Location 6
6.4 Focus group/interviews and creative activity method 6
6.5 Interview/focus group discussion guide 7
6.5 Data analysis 8
7. Ethical considerations 8
7.1 Study procedure benefits 8
7.2 Study procedure risks 8
7.3 Confidentiality and privacy 9
7.4 Data storage and record retention 9
7.5 Regulatory considerations 9
8. Early termination (if applicable) 9
9. Conflict of interest 9
10. Funding 9
11. Research outcomes 10
12. References 10
Appendix 1 - CHA PREMS Steering Committee 11
Appendix 2 - Overview Next Steps in CHA Paediatric PREM Development Process 12

NOTE: SWSLHD recommends getting a Peer Review prior to submitting the ethics application and disclosing any conflicts of interests in the protocol. Please see the *Peer Review Guide and the Disclosure of Interests and Management of Conflicts of Interest Guide, which support the implementation of the Australian Code for the Responsible Conduct of Research (the Code).*

1. SUMMARY

**Study Title**	Understanding what matters to children and young people when visiting hospital? The Development of a National Paediatric Patient Reported Experience Measure
**Aims/Objectives**	Primary:<br>The project objectives are to:<br>1. Explore children and young people's perspectives on their experience of being in hospital on an inpatient, outpatient or emergency department basis in a public hospital in Australia (Stage 1)<br>2. Identify what children and young people identify as the most important factors that should be considered when evaluating children's experience of care in hospital settings (Stage 1)<br>3. Understand from the perspective of children and young people how they would like to provide feedback on their hospital experience<br>4. Develop a list of questions that can measure children and young people's experience of care in hospital inpatient, outpatient and emergency care settings (Stage 2)<br>5. Evaluate 'Paediatric PREM' to determine validity and reliability (Stage 3)

	6. Evaluate acceptability, feasibility, and effectiveness, as well its implementation into clinical practice (Stage 4&5)
**Study design (Stage 1)**	Qualitative cross-sectional study using face-to-face or virtual focus groups, interviews or take-home creative activity with children and young people.
**Planned sample size**	We aim to interview ~30-50 Children and young people, or until we reach data saturation.
**Inclusion criteria**	We will recruit children aged 6-12 and young people aged 13-18 who have attended a participating hospital in the past 3 months.
**Study procedures**	Children and young people will be offered the opportunity to participate in focus groups, interviews or a take-home creative activity while attending a participating hospital or health service. Parental consent will be sought for all children and young people under 17 to participate. Focus groups will take ~45-60 minutes.
**Analysis considerations**	The research will be recorded, transcribed verbatim, and then analysed using NVivo 11, coding line-by-line. Content and thematic analysis using an inductive approach will be used to identify themes.
**Study duration**	12 months

2. BACKGROUND AND RATIONALE

Patient Reported Experience Measures (PREMs) allow health services to understand how care is experienced by children, young people, and families/carers by collecting feedback on their views. International evidence suggests this helps services to identify strategies and models of care that enhance experience and outcomes of care for children, young people, and their families/carers.

Children's and young people's rights to be heard in healthcare services are of vital importance as significant discussions and decisions take place in this setting about health and treatment options which have a direct impact on their current and future wellbeing. The rights of children and young people to be heard in matters that affect them are enshrined in Article 12 of the United Nations Convention on the Rights of the Child (UNCRC) (United Nations, 1989), and in the Charter of Children's and Young People's Rights in Healthcare Services in Australia (Children’s Hospitals Australasia & Association for the Wellbeing of Children in Healthcare, 2011).

In the Charter of Children’s and Young People’s Rights in Healthcare Services, these manifest in:

1. The right to express their views, to be heard and taken seriously, and
2. The right to participate in decision-making and, as appropriate to their capabilities, to make decisions about their care.

These include the right to be heard and taken seriously at a policy level. Children’s and young people’s views should be sought when making decisions about healthcare and how to improve their experience of healthcare. This discussion needs to be held in a way that is appropriate for children at their developmental level and recognises that the engagement process with children for this consultation varies greatly from methods that work with adults (Children’s Hospitals Australasia & Association for the Wellbeing of Children in Healthcare, 2011). These rights are echoed in Article 12 of the UNCRC where they declare that: “State Parties shall assure to the child who is capable of forming his or her own views the right to express those views freely in all matters affecting the child”
And “…the child shall in particular be provided the opportunity to be heard in any judicial and administrative proceedings affecting the child…” (UN General Assembly, 1989).

Despite the UNCRC and Charter of Children’s and Young People’s Rights in Healthcare Services, in an audit of children’s hospitals across Australasia, it was found that the right of a child to express their views and be heard scored lowest for all of the rights on the Charter of Children’s and Young People’s Rights in Healthcare Services, alongside the right for privacy (Children’s Hospitals Australasia & Association for the Wellbeing of Children in Healthcare, 2011). This indicated that although there is a growing recognition of the importance of this in health services, in practice it is not significantly achieved.

There is also growing recognition that adolescents should be involved in treatment decision making. Adolescents may also want to make treatment decisions themselves or take part in the decision-making process (Wakefield et al., 2011; Weaver et al., 2015). Information provision that is perceived to be parent-centred can be disempowering for young people (Lin et al., 2020). However, children’s and young people’s views are often neither sought nor acknowledged in the health system (Coyne, 2008). PREMs have the ability to incorporate children’s and young people’s views as a measure allowing feedback to services about how they are progressing on these important aspects related to the rights of children and young people.

Children and young people are often excluded from interactions between their health professionals and parents and have unanswered questions about their care and illness (Schalkers et al., 2015). Many factors such as health professional’s communication styles and behaviours, fear of causing trouble by asking questions, difficulty understanding medical terminology, and parent’s actions hinder children and young people from actively seeking to be involved in consultations regarding their care (Coyne, 2008). Schalkers et al. (2015) found in their study that some children were uncertain as to whether they had a choice in their care, but all wished to engage in the decision-making process regarding their own care.

Additional barriers to participation are experienced by a range of minority groups including ethnically and culturally diverse communities, people whose primary language is a language other than English, Aboriginal and Torres Strait Islander people, people with a disability and people identifying as LGBTIQ+. Barriers experienced include power imbalances, both overt and unintended discrimination, and a lack of respectful and culturally appropriate communication. In addition, some minority groups experience economic barriers and language barriers which have been demonstrated to impact on health outcomes. It is therefore important that the needs of minority groups who can experience a range of barriers are consulted to understand their perspective on how care in hospital is experienced.

PREMs provide a mechanism for clinicians, service providers and commissioners to use to evaluate current care and to shape future service provision (Gore et al., 2016). For example, the Australian Hospital Patient Experience Question Set (AHPEQS) is a nationally consistent tool available in multiple languages which surveys patient’s experience of care in public or private hospitals in Australia (Australian Institute of Health and Welfare, 2018). However, there is currently no nationally available tool validated for use for children and young people in the Australian healthcare setting.

One of the key issues with current PREM tools in paediatric populations is reduced uptake of these tools by children, young people, and their hospitals. Research conducted for children and young people, by children and young people, has strong potential to increase the effectiveness of measurements tools such as PREMs (Thomson et al., 2020; McNeill et al., 2021). By involving children and young people in developing measurement tools such as PREMs, they can also provide suggestions on how to increase the uptake of these tools in children and young

people in hospitals (McNeill et al., 2021). Furthermore, by collaborating with children and young people in selecting measures to assess experience of care, there are opportunities to enhance the relevance of measurement tools, thus enhanced capacity to generate more meaningful data (McNeill et al., 2021).

A study conducted on the effectiveness of six PREMs tools for children and young people in the United Kingdom identified some limitations of the tools such as language barriers, cultural relevance and accessibility for those with learning disabilities (Wray et al., 2018). In Australia, a similar review of existing PREMs for children and young people identified that a small number of PREMs exist, which are long, not validated for use in Australia or require a subscription to utilise. Another key challenge to the use of current PREM tools in paediatric populations is the completion of these tools by parents. Schalkers et al. (2015) confirms that parents or legal guardians are generally invited to evaluate the care and services of paediatric hospitals rather than the children themselves. This is problematic for the use of PREM tools in the paediatric populations as the views of parents, although important, cannot represent those of their children (Schalkers et al., 2015).

Other key issues with current PREM tools are the lack of standardisation, validation, and translatability of these tools across hospitals, which limits the comparison of data and improvement of care through recommendations. The standardisation and validation of PREM tools is important to ensure that the surveys developed will evaluate children and young people's experiences of care in hospitals and generate meaningful data (Kaitelidou et al., 2019). The translatability of these tools is also important as it allows for quality improvement and comparisons across hospitals (Aoki et al., 2020).

It is anticipated that a paediatric PREM tool validated and tested in the Australian hospital setting will increase uptake. This will allow hospital services to survey children and young people to evaluate the care provided from the perspective of children and young people. Developing a national tool will increase consistency of approach, allow sharing of data on outcomes from the perspective of children and young people, and increase adherence to the rights of children and young people to be heard in healthcare services.

3. STUDY AIMS/OBJECTIVES

The project objectives are to:

1. Explore children and young people's perspectives on their experience of being in hospital on an inpatient, outpatient, or emergency department basis in a public hospital in Australia (Stage 1)
2. Identify what children and young people identify as the most important factors that should be considered when evaluating children and young people's experience of care in hospital settings (Stage 1)
3. Understand from the perspective of children and young people how they would like to provide feedback on their hospital experience
4. Develop a list of questions that can measure children and young people's experience of care in hospital inpatient, outpatient, and emergency care settings (Stage 2)
5. Evaluate 'Paediatric PREM' to determine validity and reliability (Stage 3)
6. Evaluate acceptability, feasibility, and effectiveness, as well its implementation into clinical practice (Stage 4)

<u>This protocol outlines Stage 1 only</u>. Stage 2 and 3 will be determined based on the findings of Stage 1. Ethics and governance amendments will be obtained prior to Stages 2 and 3 being conducted.

4. PARTICIPATING SITES

Women's and Children's Hospital, SA,
Sydney Children's Hospital Network, NSW
John Hunter Children's Hospital, NSW
Queensland Children's Hospital, QLD.
Royal Darwin Hospital, NT

5. STUDY DESIGN

5.1 Study Type

Stage 1 is a qualitative cross-sectional study using face-to-face or virtual focus groups, interviews, or a take-home creative activity to gain the perspectives of children and young people. The study involves:

- Completing the consent form and several demographic questions alongside the consent form
- Attending a focus group, interview or completing and recording a take-home creative activity

5.2 Expected Study Duration

The study will begin once ethics and governance approval has been obtained, aiming to commence conducting focus groups/interviews in the first half of 2022. Following analysis of Stage 1 data, we will submit an ethics and governance amendment to conduct Stages 2 and 3 (Table 1).

Table 1 Study Gantt chart

	2021				2022			
**Task**	**Q1**	**Q2**	**Q3**	**Q4**	**Q1**	**Q2**	**Q3**	**Q4**
Establishment of steering committee								
Finalisation of interview script + process map								
Ethics								
Stage 1 recruitment								
Stage 1 focus groups/interviews								
Stage 1 data analysis								
Stage 1 manuscript preparation								

5.3 Data Source and Population

Face-to-face and virtual focus groups and interviews will be recorded with consent of caregivers and assent of children and young people. To provide alternative means of capturing the voice of younger children when face-to-face interviews may not possible due to infection control risks, a take-home creative activity will enable the voice of young children to be included. Children will be asked to draw about their hospital experience and a parent/guardian will be asked to video record their child explaining their drawing and describing what matters to them as a patient in a hospital.

Table 2 Population variables

Participant Variables	
Demographic information	
Sex	Male, Female, Other, Do not wish to respond
Age Child or young person	6-18 years
Aboriginal or Torres Straight Islander background	Y/N/ Do not wish to respond
Main language spoken at home	English or other (specified)
Hospital admission information	
Number of hospital visits in the last 12 months	Number inpatient admissions (last 12 months)
	Number of outpatient visits (last 12 months)
	Number of ED visits (last 12 months)
Barriers to participation in hospital	Does your child have any significant physical or intellectual disabilities that impact them that you would like to share with the research team?

5.4 Recruitment and Screening

Invitations to participate will be disseminated in person by Starlight Children's Foundations team members via the distribution of flyers, posters and via social media advertisement (e.g. Twitter, hospital website or social media page).

In South Australia flyers will be displayed in ward areas by WCHN staff, WCHN staff will be encouraged to offer participation to children and young people and obtain a ‘consent to contact' from the WCHN parent or guardian before they can be approached by the non-WCHN study personnel. Following receipt of Consent to Contact forms Starlight Foundation staff will contact parent or guardian by their preferred contact details to explain the study and see if they would like their child to be involved in the study

Consent will be obtained from the parent or young person (17years or older) prior to the scheduled focus groups or interviews.

The focus group consent form will ask participant demographic information and details of recent hospital admission to confirm eligibility to participate. Name, phone and email contact details for parents will be requested to enable scheduling and ensure parents can be contacted to support the young person if required. Young people 17 years or over will be able to sign their own consent form

Children and young people under 17 years old will also complete a non-binding assent outlining the expectations of participation in line with the research by Hoppe et al. (1995) and Morgan et al. (2002).

Participants known to experience barriers to participation in healthcare will be sought, including those who experience language, cultural and economic barriers including those with disability. It is therefore anticipated that ethnically and culturally diverse communities, people who speak a primary language other than English, Aboriginal and Torres Straight Islanders and people with a disability may form part of the cohort of children.

Where necessary additional communication strategies (assisted communication strategies, interpreters or parental support) will be employed to ensure the ‘voice' of children known to experience barriers to participation is included.

5.5 Inclusion Criteria

All children and young people between the ages of 6 and 18 years who are either a) currently at a participating hospital or b) have attended a participating hospital, including as an outpatient or to emergency department within the last 3 months at the time of the consent form being returned are eligible to participate.

Should a child wish to be involved but require translator services, the researcher will inform the study coordinator who will arrange a translator to be available face-to-face or via telephone at a time suitable for the family to complete study participation.

Table 3

Inclusion criteria
Age: 6-18 years
Has attended a participating hospital in Australia within the last three months at the time of the consent form being returned

5.6 Exclusion Criteria

Table 4

Exclusion criteria
Age: under 6 years or over 18 years
Have not attended an Australian hospital in the last three months at the time of the consent form returned
Parent/guardian is unable or does not wish to provide proxy consent for their involvement
Children who do not provide assent for their participation

5.7 Consent Process

1. Invitations to participate will be disseminated in person through Starlight Children's Foundations team members via the distribution of flyers, posters and via social media advertisement e.g. Twitter, hospital website or social media page.
2. Parents/guardian or young person 17yrs or older who express interest in participating in person or via email will be provided with a Parent _Guardian Participant Information Sheet, a Child or Young person Participant Information Sheet, a consent form for the parent and an assent form for the child. This includes questions to establish eligibility criteria, withdrawal processes and preferred method of contact.
3. Parent/guardian or young person will be offered the ability to participate in person or virtually.
4. Parents/ guardian will be asked to nominate if they would like to be present during the interview/ focus groups.
5. Consent forms can be returned by parents/guardian in person to Starlight team members (at the hospitals' Starlight Express Room) or via email to coordinating principal investigator prior to the focus group or interview.
6. Consent forms and assent forms will be scanned and saved in secure files and hardcopies destroyed.
7. Date and time of focus groups/interviews will be determined based on availability of participant and researchers. If a child or young person is unable to attend on the day an alternative time will be sought.
8. Participants who were emailed but have not consented or declined participation will be sent a follow-up email ~2-3 weeks following their initial invitation email. If they still do not respond, a third final email will be sent ~2-3 weeks later at which point they will be considered lost to follow-up.
9. Follow up: We will send participants a reminder email regarding their focus groups/interviews a day prior,

6. Study Procedures

6.1 Steering Committee

We have convened 18 professionals with a variety of relevant clinical and research backgrounds to comprise the steering committee for the CHA Paediatric PREM Question Set project. The role of the steering committee is to provide direction and expert input into the design of this project through stages 1-3.

See Appendix 1 for steering committee and qualifications.

6.2 Focus Group/Interview Composition

Table 5

Group Composition	Age – groups stratified by age – aiming for a maximum of 2 – 3 years age difference
	Size – 5-8 participants in each group
	Gender – heterogenous with approx. 50:50 male to female ratio
	Geographical region – naturally stratified by geographical area and nearby hospitals
	Adults present – 2-3 adults per group to facilitate discussion and run focus group

Groups will include 2-3 adults which will cover: 1 moderator, 1 recorder/observer and in instances where there are primary-aged children 1 designated adult to take children to the bathroom, manage any behavioural challenges that may arise as well as any unexpected issues that may arise throughout.

Individual interviews, online focus groups or take home creative activity will be offered to any child or young person with travel restrictions caused by disability, government travel restrictions, living arrangements, location, parental travel limitations or due to other physical and/or developmental needs of the child.

6.3 Location

Focus groups will take place in the Starlight Express room in participating hospitals. Focus groups or interviews may also be conducted online via Microsoft teams, using an authenticated email address to ensure participation is limited to invited participants. Interviews may be conducted in Starlight Express rooms, in individual patient rooms, or online via Microsoft Teams.

6.4 Focus group/interviews and creative activity method

Information will be collected using one-off focus group discussions or interviews led by a moderator or via take home creative activity.

All the focus group discussions, interviews and creative activity will focus on obtaining respondents' views about their hospital experience and what is meaningful to them, following the discussion guide outlined below. Beyond the general structure of the focus group/interviews and their common questions and themes, each group will be tailored to the group members' age and understanding. For example, the focus group/interviews with primary school aged children will use play or art-based approaches and a limited number of open-ended questions in age-appropriate language; and the focus group/interviews with young people in high school will be oriented towards a group discussion, guided by a discussion plan involving more open-ended questioning.

The take home creative activity aims to enable children the opportunity to share their view in a virtual (COVID safe) space more suitable for children than traditional online interviews. Children will be sent a craft pack and accompanying video. The video will have captain starlight asking them to draw about their hospital experience. Parents will be asked to video their explanation and upload via secure platform.

Focus group/interview duration will be 45-60 minutes and group size will be 3-5 participants. Prior to the focus group/interview participants will be contacted with additional information regarding set up and will participate in a 1-minute trial of the platform with the researcher to ensure they are able to connect and use the platform.

General Structure of focus group/interview session:
Figure 2

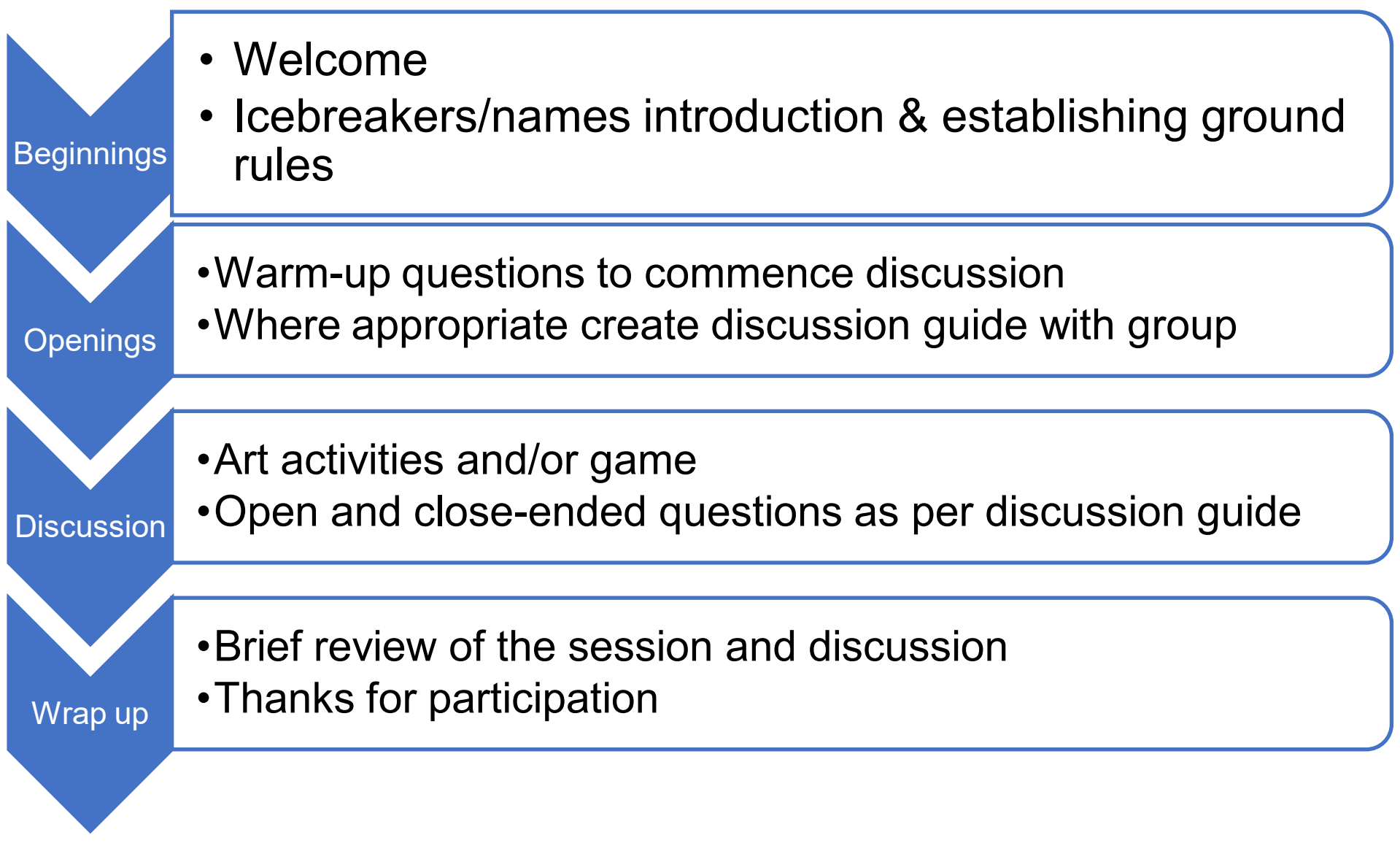

6.5 Interview/focus group discussion guide

1. Imagine the best hospital experience that you can (/ A Perfect Hospital).

- What is like when you arrive at the hospital?
- What does it look like?
- What are the doctors/nurses like?
- How do people talk to you/ speak with you?
- How involved were you in discussions that affect you?
- How involved were you parents and careers in decisions?
- Were your parents involved as much as you wanted
  Further prompt - Why is that good, or important.

2. Imagine the worst hospital experience that you can

- What is like when you arrive at the hospital?
- What are the doctors/nurses like?
- What is that environment like?
- How do people talk to you/ speak with you?
- How involved were you in decisions that effect you?
- Why is that bad, / how does that make you feel when that happens

For older children i.e. > 12 years (or younger if assessed to be developmentally appropriate)

3. Provide an overview back to the group/ person of the key things discussed and ask participants which are the most important to you that hospitals get right

- Ask if anything has been missed that is important?
- Of all the things mentioned which are the most important to you that hospitals get right?

4. **How would you like to provide feedback?**

<u>Young person > 12 years</u>
Hospitals want to know about your experience so they can make it better for others who need to be cared for in a hospital

Would you want to provide feedback about your experience when you visit?
How would you prefer to provide feedback to the hospital on your experience of being in hospital?
Prompts

- Would you complete an emailed survey?
- Would you be happy to receive a text message with a link?
- Would you provide feedback on a phone or a Tablet?
- Would you like to have somebody speak with you, just like we are now?
- QR code on wall,
- Interactive avatar-based game.
- What else can you imagine as being the best way to provide feedback to hospitals?

<u>Child - Less 12 years</u>

Would you want to provide feedback about your experience when you visit?
How would you prefer to provide feedback to the hospital on your experience of being in hospital?

- Would you like to answer questions written on a piece of paper? I.e. Survey
- Would you answer questions on your phone or your mum's or dad's phone?
- Would you play a game on phone or tablet that describes your experience?

- Would you like to have somebody speak with you just like we are now?
- What else can you imagine as being the best way to provide feedback to hospitals?

What questions should we ask in a patient feedback survey

Hospitals want to make visits better for kids, to help us know how to do this, what questions do you think people working in hospitals should ask kids when they visit to ensure they can make things better?
E.g. If you were in charge of this hospital what do you think we should be asking kids about their stay?

Young person
We want to create a survey which asks about your experience at hospital. What should we include in the survey?
E.g. If you were the boss of this hospital what would you want to know from the kids coming here to make it a better experience ?

6.6 Data Analysis

Focus group and interviews will be video or audio recorded and transcribed verbatim. If video recorded, a second moderator will complete summary of the interaction to capture non-verbal interactions using a predetermined template.

For the take home interactive craft activity parents or guardians will be asked to video or audio record their child explaining their drawing or writing and describing what matters to them while a patient in a hospital. If video recorded, the researcher reviewing the video will also complete an additional summary of the art and any non-verbal interactions using the same predetermined template.

The MP4 audio file of these videos will be used to create a verbatim transcript. On receipt of the video or audio file from the hospital site, the file will be renamed at CHA with a unique identification number, deidentifying the original audio file. The Otter.ai platform may be used as a transcription tool to produce the transcript of the audio file. Each transcript and audio file will be reviewed and checked for consistency and accuracy at CHA, or by a designated project member.

The transcripts will be analysed using NVivo 11, coding line-by-line. Thematic analysis using an inductive approach will be used to identify aspects of care that are important to children and young people. This will involve developing a coding tree that explores the common responses or themes emerging from the focus groups/interviews. We will analyse data by age and hospital setting (ED, inpatient, outpatient) to identify any variance between groups.

7. ETHICAL CONSIDERATIONS

7.1 Study Procedure Benefits

Participants will receive no direct benefits from their participation in this study. However, their responses will inform the research in this area and ultimately, we expect the questionnaires developed through this research to improve children's experience of care.

7.2 Study Procedure Risks

There is a possibility that study participants may experience distress discussing their past experiences of care in hospital. Staff facilitating focus groups will be responsible for the well-being of participants and will be encouraged to monitor for signs of distress or sharing of content that is potentially upsetting to others. Facilitators may advise a participant that a topic may be upsetting to others and suggest an individual discussion of these matters eg Discussion around death or dying. Should a child or young person become distressed by the nature of the questions during the focus groups/interviews, they will be advised that they do not have to answer questions and may stop the interview at any time.

If children or young people are upset by their involvement support will be provided. This may include, contacting parents to advise them of distress and encouraging them to provide emotional support. Providing contact details for mental health services e.g., Kid's Helpline or facilitating follow up with hospital pastoral and spiritual care services team or other relevant counselling staff responsible for providing emotional support and counselling at the relevant hospital (pending on the severity of expressed emotion).

Contact numbers have been provided in the participant information sheet for LifeLine, Kids Helpline and Mental health access line, as well as providing emergency access number 000.

In some cases, a child may disclose information which must be reported. This includes information about a current risk to the child such as abuse, or a risk they were previously exposed to. In the event that information of this nature is disclosed during focus groups or interviews, the facilitator will inform the principal investigator who will proceed with standard risk management and mandatory reporting guidelines as per hospital policy.

Another risk is the possibility of a child feeling coerced into voicing certain opinions at the focus groups/interviews. This risk is augmented by the fact that the focus groups/interviews are being held in a non-clinical setting by Starlight Express staff skilled in management of focus groups/interviews and rapport with children.

Risk management procedures will also be incorporated into online focus group discussions/interviews. A parent/guardian will also be encouraged to be in attendance or available nearby for virtual focus groups/ interviews for children up to 16 years of age. Participants and facilitators will be instructed to consider other participant privacy by encouraging the use of headphones or utilising a private space in the house. Staff facilitating focus groups will be responsible for the wellbeing of participants and will be encouraged to monitor for behaviour that is inappropriate or potentially upsetting to others e.g. offensive conduct will not be tolerated and people may be excluded from participating following warnings if required.

7.3 Confidentiality and Privacy

Only the research staff directly involved in the study will have knowledge of and access to participants' data. Any identifiable information that is collected in relation to this study will remain confidential and will be disclosed only with participants' permission, or except as required by law. Interview data will only be labelled with a unique identification number. To maintain privacy and confidentiality, these video or audio recordings will only be accessible to the relevant members of the research team and will be stored securely in the cloud under CHA's Microsoft Azure tenant as described in section 7.4 below.

Confidentiality and privacy of discussions will be maintained through standardised protocols. Parents/guardians and participants will be requested not to share information about their peers outside of the focus group discussions. Online participants will be asked to ensure that no one else is in or near the vicinity of the room during the virtual discussion. Researchers will implement security measures by using up-to-date security software, secured wi-fi networks and videoconferencing platforms approved for clinical and corporate uses. Participants and parents/guardians will also be informed of security measures they can undertake such as accessing the discussion from a private email and a private room.

7.4 Data Storage and Record Retention

All paper data will be stored in a secured cabinet located in investigator offices (e.g. hard-copy consent forms, family contact number), until transferred to electronic records, and then securely destroyed in line with hospital protocols.

Electronic data including audio recordings and video's will be stored securely in the cloud under CHA's Microsoft Azure tenant in an on-shore data warehouse. MS Azure's compliance and certification against Australian standards is available here.

Access to project data is available only to approved research project staff via an encrypted VPN connection to MS Azure resources. Identity management for staff is provided by Microsoft, and accounts are protected with multi-factor authentication (MFA). Access to stored data will be monitored and managed internally by the CHA IT team.

When external access to files needs to be granted (eg transfer of audio, video files from participating sites), access will be granted subject to the same management conditions as research staff access. Accessible only by encrypted VPN connection to the CHA MS Azure tenant.

In the case where passwords need to be provided to researchers, the password sharing tools from our password management software will be used to distribute passwords in a secure manner that can be monitored by the CHA IT team.

Videos will be destroyed following analysis with audio recording and transcripts stored for 33 years after research project completion. Following this time, all electronic files will be permanently deleted from the server. All text messages and call logs will be immediately deleted following contact.

7.5 Regulatory considerations

Compliance and regulatory guidelines

This study will be conducted in compliance with the:

- The National Health and Medical Research Council National Statement on Ethical Conduct in Human Research,
- The Australian Code for the Responsible Conduct of Research, and
- Declaration of Helsinki.

Ethical review

Prior to the commencement of the study, the protocol, Informed Consent form, information sheet will be submitted to the south-west Sydney Human Research Ethics Committee (HREC) and relevant governance committee.
During the study, the investigator will submit to the HREC the following:

- amendments to the protocol,
- serious and unexpected adverse events and the outcome,
- specific site updates as agreed to by the investigator and respective HREC, and
- any additional information (e.g., unexpected serious adverse events reported by other sites, amendments to the Investigator Brochure, and administrative changes to the protocol).

8. Early Termination (IF APPLICABLE)

Withdrawal process

This study is completely voluntary, and participants can withdraw from the study at any time. Participants and parents of participants will be able to choose to withdraw from the study at any time with no consequence to the medical care they receive or relationship with the hospital or associated organisations. Due to the focus groups/interviews being recorded as a collective discussion it will not be possible to withdraw already collected data without compromising the data of other participants. However, individual comments can be removed from the written transcripts.

9. CONFLICT OF INTEREST

None, free for all to use.

10. FUNDING

Funded by Children's Healthcare Australasia with in-kind contribution of Starlight Children's Foundation

11. RESEARCH OUTCOMES

We plan to share the results of the study with the Human Research Ethics Committee (HREC) for ethical monitoring purposes as well as in peer-reviewed journals, at conferences or other professional forums for the promotion of knowledge in the health services. When published, information will be provided in such a way that participants cannot be identified. There are no existing contractual or other agreements or obligations with other parties that may influence the publication plans or the sharing of data. All participants will be emailed a copy of the research findings.
The data collected in Stage 1 of this process will be used to inform the research in Stage 2 and Stage 3 as outlined in the summary and in the study aims/objectives. The final PREM tool developed will be made freely available on the CHA website for open use.

12. REFERENCES

Aoki, T., Yamamoto, Y., & Nakata, T. (2020). Translation, adaptation and validation of the hospital consumer assessment of healthcare providers and systems (HCAHPS) for use in japan: A multicentre cross-sectional study. BMJ Open, 10(11), e040240-e040240. https://doi.org/10.1136/bmjopen-2020-040240

Australian Institute of Health and Welfare. (2018). *Australia's health 2018.* Canberra: AIHW.

Children's Hospitals Australasia, Association for the Wellbeing of Children in Healthcare. (2011). *Charter of Children's and Young People's Rights in Healthcare Services in Australia.* Ronald McDonald House Charities.

Doswell, W.M and Vandestienne, G (1996), *The Use of Focus Groups to Examine Pubertal Concerns in Preteen Girls: Initial Findings and Implications for Practice and Research,* Issues in Comprehensive Pediatric Nursing, 19(2), 103–120. doi:10.3109/01460869609038051

Gore, C., Griffin, R., Rothenberg, T., Tallett, A., Hopwood, B., Sizmur, S., O'Keeffe, C., & Warner, J. O. (2016). New patient-reported experience measure for children with allergic disease: Development, validation and results from integrated care. Archives of Disease in Childhood, 101(10), 935-943. https://doi.org/10.1136/archdischild-2015-309598

Gibson, Faith (2007), *Conducting focus groups with children and young people: strategies for success*, Journal of research in nursing Sage Publications, DOI: 10.1177/

Hoppe, M. J., Wells, E. A., Morrison, D. M., Gillmore, M. R., & Wilsdon, A. (1995). *Using Focus Groups to Discuss Sensitive Topics with Children. Evaluation Review, 19(1), 102–114.* doi:10.1177/0193841x9501900105

Kaitelidou, D., Economou, C., Galanis, P., Konstantakopoulou, O., Siskou, O., Domente, S., de Boer, D., Boerma, W. G., & Groenewegen, P. P. (2019). Development and validation of measurement tools for user experience evaluation surveys in the public primary healthcare facilities in greece: A mixed methods study. BMC Family Practice, 20(1), 49-49. https://doi.org/10.1186/s12875-019-0935-6

Lee, Siew Pien & Nurasikin, Mohamad. (2018). Children's Experiences in Health Care Decisions: An Overview. International Journal of Care Scholar, 1(2), 37-41.

Lin, B., Gutman, T., Hanson, C. S., Ju, A., Manera, K., Butow, P., Cohn, R. J., Dalla-Pozza, L., Greenzang, K. A., Mack, J., Wakefield, C. E., Craig, J. C., & Tong, A. (2020). Communication during childhood cancer: Systematic review of patient perspectives. Cancer, 126(4), 701–716. https://doi.org/10.1002/cncr.32637

McNeill, M., Noyek, S., Engeda, E., & Fayed, N. (2021). Assessing the engagement of children and families in selecting patient-reported outcomes (PROs) and developing their measures: A systematic review. Quality of Life Research, 30(4), 983-995. https://doi.org/10.1007/s11136-020-02690-4

Pierce, J. 2017, "Supporting the Health Care Needs of the LGBTQI Community", Journal of consumer health on the Internet, vol. 21, no. 3, pp. 297-304.

Schalkers, I., Dedding, C., & Bunders-Aelen, J. G. F. (2015). "[I would like] a place to be alone, other than the toilet" – Children's perspectives on paediatric hospital care in the netherlands. Health Expectations : An International Journal of Public Participation in Health Care and Health Policy, 18(6), 2066-2078. https://doi.org/10.1111/hex.12174

Thomson, W., Shoop-Worrall, S., McErlane, F., Lunt, L. E., McDonagh, J., Smith, A. D., Smith, N., & Cleary, G. (2020). Validation of novel patient-centred juvenile idiopathic arthritis-specific patient-reported outcome and experience measures (PROMs/PREMs). Pediatric Rheumatology Online Journal, 18(1), 1-91. https://doi.org/10.1186/s12969-020-00481-2

UN General Assembly, *Convention on the Rights of the Child*, 20 November 1989, United Nations. https://www.unicef.org/child-rights-convention/convention-text

Wakefield, C. E., McLoone, J. K., Fleming, C. A. K., Peate, M., Thomas, E. J., Sansom-Daly, U., Butow, P., & Cohn, R. J. (2011). Adolescent cancer and health-related decision-making: An australian multi-perspective family analysis of appointment attendance and involvement in medical and lifestyle choices. Journal of Adolescent and Young Adult Oncology, 1(4), 173-180. https://doi.org/10.1089/jayao.2011.0030

Weaver, M. S., Baker, J. N., Gattuso, J. S., Gibson, D. V., Sykes, A. D., & Hinds, P. S. (2015). Adolescents' preferences for treatment decisional involvement during their cancer. Cancer, 121(24), 4416-4424. https://doi.org/10.1002/cncr.29663

Wray, J., Hobden, S., Knibbs, S., & Oldham, G. (2018). Hearing the voices of children and young people to develop and test a patient-reported experience measure in a specialist paediatric setting. Archives of Disease in Childhood, 103(3), 272-279. https://doi.org/10.1136/archdischild-2017-313032

Appendix 1 - CHA PREMS Steering Committee

- **Barb Vernon**, CEO, Children's Healthcare Australasia — Chair
- **Dr Claire Treadgold,** National Manager, Research and Evaluation **Starlight** Children's Foundation
- **Rhiannon Beck,** Patient Reported Measures Project Officer, Clinical Governance Unit, Sydney Children's Hospital Network
- **Ann Dadich**, Associate Professor, School of Business, University of Western Sydney
- **Antonio Mendoza Diaz,** Conjoint Lecturer, School of Psychiatry, UNSW; Senior Research Officer, ICAMHS, SWSLHD
- **Susan Dyer**, Advanced Nurse Consultant - Clinical Practice Development, Nursing & Midwifery Clinical Practice Development Unit, Women's & Children's Health Network
- **Ben Elsey, Consumer**/ patient engagement lead, The Royal Children's Hospital Melbourne
- **Valsa Eapen**, Professor University of NSW, MBBS (India), DPM (India), DFT (UK), PhD (UK), FRCPsych (UK), FRANZCP (Aust)
- **Karen Ford,** ADON, Research and Practice Development, Centre for Education & Research, Royal Hobart Hospital and Adjunct Associate Professor, University of Tasmania
- **Adele Kelly**, Improvement Coordinator CHA, Children's Healthcare Australasia
- **Elaine McCall**, RN DN Nurse Consultant, Leader, Safe Care Programme, Starship Child Health NZ
- **Jessica Nikolovski**, Consumer Young Person, Sydney Children's Hospital Network
- **Eden Robertson**, Postdoctoral research fellow, Behavioural Sciences Unit, Kids Cancer Centre, Sydney Children's Hospital and Starlight Children's Foundation
- **Girish Swaminathan**, Senior Project Officer, Australian Commission on Safety and Quality in Health Care

Appendix 2 - Overview Next Steps in CHA Paediatric PREM Development Process

Activity	Purpose	How to explore or estimate	Actions required
**Stage 1: Item Development**			
Item generation/ concepts to measure	To identify appropriate items that measure experience of healthcare	Deductive methods: literature review and assessment of existing Person and Family Centred Care (PFCC) frameworks	• Literature review and mapping process - completed<br>• Existing PREMS children and young people<br>• Person and Family Centred Care Principles<br>• Australian Commission Quality &safety in Healthcare - Australian Patient experience questions set (AHPRQ)
	Identify items relevant for children and young people	Exploratory research using focus group discussions and interviews	Focus groups - representative age ranges, model of care, regionality<br>Partner services to run focus groups locally
	Evaluate which items are most important to children and young people	Prioritisation process – Participants asked to prioritise the two most important things<br>Factorial analysis of most important elements	Focus group participants to rate factors most important<br>Statistical analysis of factors identified to be most important
**Stage 2: Content** Validity**: Assessing if the Items Adequately Measure the Domain of Interest**			
Evaluation by experts	To evaluate each of the items for content relevance, representativeness, and technical quality	Assessment of feasibility, relevance, appropriateness (2.4 AHPEQ)<br>Questions relevant to all/ most services within the control of services and able to be improved upon	Application of feasibility and quality criteria by health professionals and QI staff in multiple states
	Finalise question to go to testing	Expert advisory group input to assess results of feedback to agree on question to proceed to testing	Collated feasibility feedback reviewed by expert panel
**Stage 3: Pre-testing** Questions**: Ensuring the Questions and Answers Are Meaningful**			
Evaluation by children and Young people	To assess the extent to which questions reflect the domain of interest and that answers	Conduct interviews with children & young people to evaluate face validity	Partner services to conduct cognitive interviews with children & young people to assess comprehensibility and interpretation of questions and response

Cognitive testing	produce valid measurements	Administer draft questions to 5–15 interviewees in 2–3 rounds while allowing respondents to verbalize the mental process entailed in providing answers	options. Consider 'think-aloud' technique to detect differences of interpretation and ambiguity
**Step 4: Survey** Administration **and Testing: Gathering Enough Data from the Right People/Gathering data from stakeholders**			
Survey administration	Gathering enough data from the right people with minimum measurement errors	Administer potential questions to representative sample, multiple states and types of care (ED, inpatient, outpatient)<br>Consider;<br>Number respondents of different age groups<br>• Method of collection - paper, plus electronic systems<br>• Literature recommended sample size is 10 respondents per survey item and/or 200-300 observations<br>• Consider time point post discharge for children<br>• Some services currently survey while children are an inpatient<br>• Use cross-sectional data for exploratory factor analysis	Tests for validity and reliability<br>• Construct validity - Factorial Analysis<br>• Convergent validity (using additional items)<br>• Content validity<br>• Reliability - Cohen's Kappa, PBA Kappa<br><br>Refinement of questions wording and number of questions to occur through development
	Test Retest validity	Use data from a second time point, 2 weeks later	Retest subset of population 2 weeks later<br>Analysis - intra class correlation coefficient or Pearson product-moment correlation
Stage 5 Survey Endorsement			
Survey distribution	Survey utilised widely within paediatric services around Australia	Seek endorsement and uptake in children's hospitals, paediatric units, State health departments	Consultation and promotion to occur throughout development process.<br>PREM to be released on public area of CHA website for free /open use.
		Seek endorsement from ACSQHC	ACSHHC participating in steering committee,<br>Development process to reflect / consider the AHPEQ process to align where possible<br>Consultation to occur throughout development.
