# Supplementary material for: Playing It Differently: Using Performance‐Based Interviewing to Engage Children in Healthcare Research
Source: Health Expect. 2026 Oct 26;29(5):e70922. doi: 10.1111/hex.70922 (PMC13700104; doi:10.1111/hex.70922)
Supplement: Supplementary file 3 [file HEX-29-e70922-s004.pdf]

Safeguarding Children and Young People
Code of Conduct Procedures

1. Introduction

These procedures have been written to provide Starlight's People Managers and Volunteer Advisors with information to help their team to apply the *SCYP Code of Conduct.* It is not an exhaustive list; rather it provides examples of how the policy can be applied in the day-to-day work of Starlight. The procedures should be read in conjunction with the *SCYP Code of Conduct Policy.*

If you have any questions please ask the National Program Delivery Managers, National Volunteer Manager, HR Talent Manager, Head of Programs or Head of People and Culture.

2. Scope

These procedures apply to all team members. Team members include our Board Directors, Executive team, People Managers, all paid team members and all programs volunteers, including interns (i.e. all volunteers interacting with children and young people).

3. Definitions

See Appendix 1.

4. Roles and Responsibilities

All Starlight team members must understand and comply with the *SCYP Code of Conduct Procedures.* All team members should also do everything they can to ensure anyone who interacts with Starlight does so in a way that reflects our *SCYP Policy* and *Code of Conduct.*

Some Starlight team members have additional responsibilities, as outlined in the table below.

Position	Responsibility
Starlight Executive Team	• Promote a commitment to these procedures and their expectations.<br>• Ensure all People Managers have access to support and advice to understand and implement these procedures.
SCYP Representatives	• Review and update these procedures in consultation with relevant stakeholders.<br>• Support the implementation of these procedures, including providing training and advice in the application of the procedures.
People Managers	• Understand the *SCYP Code of Conduct Policy* and related procedures and ensure it is followed by your teams.<br>• Ensure any *SCYP Code of Conduct* breaches are logged.<br>• Ensure your team have access to the SCYP Code of Conduct Policy Code of Conduct procedures.

Starlight acknowledges and pays respect to the traditional custodians of the lands on which we work, live and play.

5. Procedure Steps

Sexual Misconduct

Any form of sexual behaviour between, with, or in the presence of, children or young people participating in Starlight programs is always unacceptable.

- Team members must always log any attempts of sexual behaviour between children and young people, as sometimes it indicates the child or young person has experienced inappropriate sexual behaviour themselves and needs support. The log will enable the Safeguarding Children and Young People Representative to develop a response, e.g. notify the child's social worker.

Positive Guidance

Everything we do in Starlight programs is undertaken positively. Occasionally we need to provide a child or young person with positive guidance and the *SCYP Code of Conduct* helps to clarify the strategies team members may use.

- Starlight team members need to be sensitive to children and young people who have little to no control over their behaviour due to illness, cognitive impairment, or disability. Our hospital partners, a child's treating team (e.g. nurse) and/or the child's parents can help to develop appropriate ways to manage any recurring issues.

Hospital Programs

- Where a parent or guardian is present, Starlight team members should always speak with the parent or guardian first and ask for their assistance in managing the child or young person.
- The child or young person's nurse can be contacted to assist in handling the situation if required.
- Team members should have conversations about inappropriate behaviour with children and/or young people in the presence of another adult e.g. a parent, nurse or a Starlight team member.
- When discussing inappropriate behaviour with a child, young person or their family, Starlight team members should:
    - Explain the inappropriate behaviour.
    - Discuss the impact of the observed inappropriate behaviour on the program and/or on the other children, young people and/or families participating in the program.
    - Suggest alternate behaviour that would not impact program delivery.
    - Explain that continuing to disrupt the program will result in the child or young person being asked to leave the program.
- If a child or young person is asked to leave the program, the Starlight team member is to contact the person's carer or guardian to discuss this behaviour with them first. If unavailable, contact is to be made with an appropriate member of hospital staff. In the instance where adequate supervision cannot be arranged, the child or young person must remain in the program until safe supervision is arranged.

If the behaviour of a child or young person escalates (where the program is impacted or other children and team members are at risk of harm), Starlight team members should call a hospital code (i.e., grey or black) to seek assistance to manage the behaviour.

Livewire.org.au

- If a young person is having a negative impact on the chat room due to inappropriate behaviour (e.g. swearing, name-calling etc.), the chat host should first request the behaviour to stop.
- In cases where young people are not responding to requests to stop, the chat host may ban the member for the remainder of the session and notify their manager via the Livewire daily debrief form submitted at the end of each shift.

Starlight acknowledges and pays respect to the traditional custodians of the lands on which we work, live and play.

Starlight Children's Foundation

- The Program Manager will review and develop a response, e.g. a warning email to the member, phone call to parents to discuss the incident, etc.

All other Starlight Programs

- Children and young people are always with their parents (e.g. Wish Week) who will take responsibility for their child's behaviour. In the rare situation they are not, and the behaviour is disrupting or creating a danger, the team member should follow the hospital procedures, e.g. always have the conversation in the presence of another adult, etc.

Recording

- Where a child or young person has been disciplined for significantly disruptive behaviour or has been asked to leave the program, a note should be made:
  - Wishgranting: in Cosmos under the child's record.
  - Hospital programs and Planet Starlight: let your manager know, in person or via email.
  - Healthier Futures: in Cosmos under the record of the clinic where it occurred.
  - Livewire.org: under the member's profile in the general notes section of the content management system.

Working with Third Parties

- Starlight has a Memorandum of Understanding (MOU) with each Paediatric Hospital that outlines the requirements for engaging with children and young people.
- Starlight Wishgranting team members provide a SCYP brief to all third parties, in addition to a verbal brief on SCYP requirements before any contact is made with children or young people.
- Starlight Ambassadors are required to view a SCYP video before each engagement in Starlight programs where children and young people are present.
- Program team members send organised guests of the Starlight Express Rooms a brief that outlines expected behaviours whilst in the program, including their SCYP responsibilities before their visit
- Chat guests receive a brief before engaging with members. Also, they are always supervised on the site by chat hosts, and transcripts are reviewed and retained for 6 months.
- Any contractors visiting the Starlight Express Room for maintenance or repairs are accompanied by a Starlight Program Manager or team member.

Professional Role Boundaries

Staying within our clearly defined roles, and the overall purpose of Starlight programs helps ensure the safety of children, young people, Starlight and you.

- All team members working directly with children and young people regularly participate in professional boundaries training; this helps to ensure they are aware of boundaries and have management strategies in place.
- Team members must be proactive with each other if a boundary is crossed, or may have been crossed, or have any concerns about professional boundaries or feelings/actions towards a particular child, young person or their family.
- Concerns must be raised with their colleague (e.g. at the end of shift debrief) and/or with their manager.
- An SCYP log should be made when a professional boundary is crossed, including when it is a 'grey' situation.
- Team members must notify their manager if they have an existing relationship with a child, young person or family who engages with Starlight. An SCYP log must then be completed to declare this pre-existing relationship and approved by the National Program Manager, Head of Programs.

Promoting Diversity, Equity, and Inclusion

We value cultural safety for all children, young people and their families, a welcoming and inclusive environment ensures everyone has a positive experience.

Team members are responsible for ensuring an inclusive culture, including paying particular attention to the needs of Aboriginal and Torres Strait Islander children and young people, as well as children and young people with a disability and/or from culturally and linguistically diverse backgrounds.

Starlight team members participate in Aboriginal and Torres Strait Islander cultural awareness training as part of Starlight's Reconciliation Action Plan, with additional training provided for team members working in our Healthier Futures Initiative.

- Team members should let their manager know if at any time they need more support to provide a culturally safe environment (e.g., training about the needs of diverse groups or cultural groups), as we have access to a wide range of resources at each hospital.

Working in Pairs

Working as part of a team helps to ensure the highest quality experiences for children and young people while also enhancing their safety and the safety of Starlight team members.

General

Starlight team members always work in pairs when interacting in person with children, young people and their families, the only exceptions those listed in the *SCYP Code of Conduct Policy* and outlined below.

All telephone and email communication with Starlight families must be recorded in Cosmos. This includes volunteer communication with families shared with the Family Relationship Coordinator (FRC) upon completion of a task.

Visits by a Family Relationship Coordinator (FRC) or Programs Volunteer to children and young people to help develop and present their wish

- Starlight team members, including programs volunteers, may be alone with a Starlight family when developing or presenting a wish, e.g. attending a celebrity wish to help manage the experience or as part of Get Connected
- Team members must be fully compliant with Starlight's SCYP (e.g. current checks, training completed, awareness of the Code of Conduct). There are no exceptions.
- During these interactions, Starlight team members are not responsible for supervising the children or young people; this is the responsibility of the parent/guardian.
- Ensure there is always a parent/guardian present when carrying out the interactions.
- If you find yourself in an unplanned situation where you are alone with the child, respectfully and sensitively, in the first instance, find the parent/guardian and speak with them about supervision.
- These interactions must be recorded in Cosmos under the child/family record, including the date and time of the visit, purpose and who attended.
- Sometimes this will require the team member to visit a family home; the home visit guidelines outlined below will apply.

Livewire.org Community Moderation with one chat host

- All site activity is captured in chat and private message transcripts and reviewed daily by the Livewire team.

- Any concerns must be escalated to the Program Manager for action.
- Actions taken are recorded in the notes section of the Livewire member's record or kept on the team member's file with People and Culture.

A hospital request for a Starlight team member to work alone, e.g., visiting a child in isolation who has a restricted visitor list, or a request for an external third party to work alone with a family

- The team member must seek approval from the People Manager and submit an SCYP log outlining the request and the ways risks will be managed. The risk must be overseen by the People Manager and SCYP Representatives.
- If a one-off request is made at short notice and prior approval isn't possible, a team member is required to advise their Manager; if the Manager is not available, a team member is required to notify a colleague and report an SCYP log on the same day of the visit.

Media opportunities

- One Starlight team member may be present where there are other contractors involved (e.g. a videographer or media outlet) and the media opportunity is recorded in Cosmos under the family record.

Research and Evaluation activities

- One Starlight team member maybe with a Starlight family when undertaking research and evaluation consultations.
- These consultations must form part of a research and evaluation project approved by the National Manager Research and Evaluation.
- Team members must be fully compliant with Starlight's SCYP (e.g. current checks, training completed, awareness of the Code of Conduct). No exceptions.
- During these interactions, Starlight team members are not responsible for supervising the children or young people; this is the responsibility of the parent/guardian.
- Ensure there is always a parent/guardian present when carrying out the interactions.
- If you find yourself in an unplanned situation where you are alone with the child, respectfully and sensitively, in the first instance, find the parent/guardian and speak with them about supervision.
- These interactions must be recorded in Cosmos under the child/family record, including the date and time of the visit, purpose and who attended.
- Sometimes these activities will require the team member to visit a family home and the home visit guidelines outlined below apply.

Home Visit Guidelines for Team Members

Team members should be aware of, and apply, the following guidelines before a home visit:

- A home visit is at the family's discretion, e.g. if the family prefer to meet face-to-face and their home is the most convenient for them. Families should always be given the option to meet in a public place convenient to them, e.g. in a café, at a Starlight office, at the hospital etc.
- The home visit must be recorded in Cosmos, including the date of visit, purpose and who attended. A home visit report is reviewed monthly by the National Program Manager
- Before the visit discuss the purpose and length of the visit with the family (generally each visit should not take any longer than 90 minutes).
- For team members, you should always email or text your manager before a home visit to let them know your whereabouts and how long the visit will be. Make sure you let them know when the visit is over.
- For volunteers, you should always let either a family member or friend know your whereabouts and expected time of return. Also, provide them with the Starlight after hour's number in case of emergencies. You should also make sure you let the family member or friend know when the visit is over.
- Always have your Starlight ID on you.

- Be careful with your safety:
  - Make sure you can exit quickly if needed, e.g. do not park where you can be blocked in, keep your car keys and mobile phone on you. When first entering the home, scan the layout to understand your exit options.
  - If there are dogs or other pets which concern you, be assertive about ensuring they are secured and pose no threat to you.
  - Be wary of external and internal trip hazards, electrical hazards, e.g. such as steps or lifted floor coverings, cords, etc.
  - End a home visit if you feel uncomfortable or risk is identified. Always err on the side of caution, e.g. if you have even the smallest 'niggle' it is best to remove yourself and let your Family Relationship Coordinator or Manager know.
- A parent or guardian must always be present and in sight during the home visit and ideally part of the conversation. If they prefer not to be part of the conversation, ensure they are within easy earshot, e.g. it would be acceptable for them to be making dinner while you meet the wish child at the kitchen table; it wouldn't be acceptable for them to be in the back yard while you were in the lounge room.
- Meetings should be held in a communal room, such as the living room or dining room. Meetings should only be held in a bedroom if the child or young person is bedbound, and you should always ask permission first. In these circumstances make sure you keep the bedroom door open and sit on a chair or the floor, never on the bed.
- If you observe anything suggesting a child or young person is at risk, make a log or immediately report it back to Starlight.

Supervision

Appropriate adult supervision helps to ensure a positive and safe environment for all children and young people.

- Never leave a child or young person alone without their parent, guardian or Starlight team member present when interacting with a Starlight program.
- Supervision should be active, e.g. in the Starlight Express Room (SER) have a clear line of vision and do not become distracted. Don't be in the back room on a teleconference.
- If you unexpectedly find yourself alone with a child, take one of the following steps and make an SYCP log before the end of your shift:
  - Move to a public area where you can be viewed by others.
  - Contact the parent/guardian.
  - Call your manager or colleague to notify them of the situation.
  - Keep any discussions non-personal.

Use of Electronic and Online Communications

A shared understanding and practices around the use of electronic and online communications help to ensure positive program experiences and reduce the risk of grooming and other unacceptable behaviour.

- The same professional standards of communication and professional boundaries apply regardless of whether this takes place in person or online/electronically.
- Communication by a
  - paid Starlight team members with families must be via Starlight phone (office or program phone, e.g. Livewire phone) Starlight or Livewire.org email, official Starlight social media, mail or face-to-face.
  - Programs volunteer must be via personal email address and phone known to Starlight and stored in Stella. Emails must be cc'd or bcc'd to the Family Relationship Coordinator, so they

can be saved to Cosmos to help effectively deliver the Starlight wish.

- A Starlight device is a phone for shared use (e.g. the Livewire mobile or office phones) and is not a mobile phone issued by Starlight to a team member for their exclusive use. For SCYP, these are considered a personal device.
- Where a Starlight device is not available, where possible, team members should be making calls using Microsoft Teams on their personal device.
- Voicemails, text messages and emails made from and to a Starlight mobile, including the Livewire mobile, must not be deleted. The Program Manager should check these messages monthly. The Livewire mobile can only be used during program delivery, e.g. team members should not take it home.
- The use of Starlight mobile phones and iPads in hospital are guided by the *Mobile Device Guidelines.* A Starlight team member (except for programs volunteers) should never respond to an email or message sent by a child, young person or their family member, to their personal email or contact number. Contact should always be made from a Starlight device, email address etc.
- Where appropriate to a positive program experience, and if supported by the young person's parents, a Starlight team member may engage in direct electronic communication with a young person over the age of 12 years. Wherever possible and appropriate, email and text messages sent to a young person aged under 18 years should be copied to their parent or guardian.

- For Livewire Online:
    - SMS/text messages are only sent via BurstSMS and reviewed weekly by the Program Manager
    - Instagram Direct Messages only to be used for purpose of Instagram content and reviewed weekly by the Program Manager
        - All communication such as phone calls via Microsoft Teams, emails and Instagram Direct Messages with a child or young person, or family member concerning the child or young person is to be recorded on the child's contact record in Cosmos.

Giving Gifts, including Wish Embellishments

Gifts are a positive part of Starlight program experiences and being clear about how and why we give gifts to children, young people and their families reduces the risk an individual could use gift-giving inappropriately, e.g. to groom a child.

Gifts must always be given:

- On behalf of Starlight, e.g. never from an individual team member.
- In an open manner, e.g. in front of parents, hospital staff, other Starlight team members etc.

Starlight team members must only give gifts in specific circumstances including:

Embellishments as part of a wish experience

- All gifts, including the cost or donated value, must be recorded in Cosmos.
- Embellishments should be mailed to the family from the Starlight office. Embellishments may also be given to a child or young person in the SER or by a Starlight team member as part of the wish presentation.

Prizes awarded for competitions, including online competitions

- For online competitions, winners must be announced online. This ensures it is recorded in the moderated transcripts. All gifts, prizes and Workshops on Demand for online competitions are recorded in Cosmos and the Prize/Gift tracking log.
    - Before sending prizes, the Program Coordinator ensures we have the correct address, plus check the "ICE last confirmed" field in Cosmos. If the ICE (in case of emergency) information is due to be reviewed in the next month, it needs to be checked and if required, updated.
- For competitions in the hospital (e.g. art competition in the SER), parents may come and collect the gift; or the gift delivered by two team members; or handed to the child or young person in the SER with a parent's verbal permission.

Prizes awarded as part of the live TV show in the SER

- All winners must be announced live on air, e.g. able to be viewed by children, parents, Starlight team and hospital staff.
- Prizes are always to be delivered by two Captains or collected by parents in the SER.

When requested by hospital staff

- For example, following a difficult procedure or celebrating a child or young person leaving the hospital after a long term stay.
- The gift must always be delivered by two team members or given to the requesting health professional to distribute.

For special occasions, including Christmas and Easter

- Gifts are only to be given at the SER party, an Easter event or otherwise, e.g. when the child is leaving the hospital for Christmas.
- There must always be two Starlight team members present when a gift is given

Gifts from celebrities and corporates when visiting the hospital

- Gifts are handed out during the event only while the celebrity is present in the SER or on the wards.
- Any leftover gifts are placed in the prize cupboard for future prizes or events.

Receiving Gifts

Families often what to show their appreciation for the support Starlight has provided them through gifts and whilst we don't expect this it is important that we respect the intention and accept the gift with grace.

Gifts must always be:

- Accepted on half of the Starlight team, even if the gift has been given to an individual.
- Shared with the team, for example consumable items such as chocolates.
- Offered to another program area if appropriate, for example a gift voucher could be used in the Moments program
- Shared with fundraising for a raffle or prize
- Logged as Donated Goods and Services if the value is over $50 and is not a consumable item

Photographs and Videos of Children and Young People

Having shared standards around photos and videos helps ensure we can continue to create great program experiences (e.g. children appearing on Starlight TV) while reducing the risk that a person may act inappropriately by photographing or using images of children or young people that could lead to perceptions of improper conduct or child abuse.

Photos of children and young people—taken by Starlight or a third party contracted by Starlight (e.g. event photographer)

- The *Starlight Consent Policy* must always be followed, i.e., a consent form must be completed before photos or videos are taken of children or young people.
- Photos/videos can only be taken on a Starlight device or by a professional photographer/film crew.
- Photos/videos taken by an approved professional photographer/ film crew, e.g. SER Christmas parties or a Starlight Wish may be edited offsite then shared with Starlight within agreed timeframes. Once shared, Starlight will request the images be deleted by the third party, and deletion confirmed.
- Starlight devices include SER/Livewire camera, SER/Livewire iPad, or a mobile phone purchased by Starlight for program purposes such as Wishgranting.

- No other device can be used—photos/videos cannot be taken on a personal camera including a mobile phone issued by Starlight for an individual to use.
- The only exception is if a Starlight device is not available or not working (e.g. camera is broken) and a team member needs to capture images or footage. In this case, they may use their device. Before taking any photos on a personal device, team members should let their manager or colleague know, and on the same day, a SCYP log is made, any footage uploaded to Sharing Impact Hub and deleted from the personal device.
- All photographs or videos taken must be saved on Sharing Impact Hub; they must not be saved on local computers, personal drives or in any other folder.
- Families may request a copy of a photo or video taken by Starlight. Team members may print out a copy in the SER, hand directly to the family, save it to a new Starlight USB for the family to keep or send a copy from the generic SER email address. Team members must never send a copy of photos or videos from a personal email address.
- When a family is involved in a PR campaign, Starlight will provide them with copies of photos and videos used. These should only be sent by the Family Relationship Coordinator or Starlight Relationship Manager and a record made in Cosmos.

Photos of children and young people—taken by families and others (e.g. in the SER)

- Team members may take photographs on a family camera, at the family's request, and of the child/ren associated with that family.
- Parents can only take photographs or videos of their children.
- If other children will be in the photo or video, consent is required from the relevant parents/carers of other children before they are included.
- External visitors (e.g. corporate visitors) cannot take photographs or videos of a child or young person.

Photos of children and young people—received from a family (e.g. a photo of their wish sent with a thank you card)

- These should only be stored on Sharing Impact Hub with any hard and soft copies securely destroyed, e.g. the email deleted.
- The photo or video can only be shared if Starlight's Consent Policy is followed, including obtaining consent from the family before sharing.

Physical Contact

It is important to have a shared understanding of what is considered appropriate physical contact at Starlight, to help ensure the safety of children and young people.

- Any physical contact with children and young people must be based on the needs of the child or young person (rather than on the needs of the team member) and must always be appropriate to the delivery of Starlight's programs.
- Providing practical, non-intrusive assistance where required is appropriate, e.g.:
  - Helping someone if they have fallen, or offering a steadying arm if they are having trouble walking.
  - If necessary to deliver the program, e.g. during face painting to steady a child's chin, ask them first if it is ok to touch their face, and tell them why you are doing so.
  - Nonintrusive gestures to comfort a child or young person who is experiencing distress, such as a hand on the upper arm or back.
  - Nonintrusive touch, e.g. congratulating a child or young person by giving a high five.
- Before touching the child or young person, seek their permission, explain what you are doing and why. The only exception is where there isn't time to do this to prevent harm to a child or others, e.g. to stop a child from falling.
- When physical contact is made with a child, it should be in response to their needs at the time, of limited duration and appropriate to their age, stage of development, gender, ethnicity and background. For example, this means a Starlight team member should never initiate a hug because it

Starlight acknowledges and pays respect to the traditional custodians of the lands on which we work, live and play.

is something they want, it must only be in response to the needs of the child or young person.

- A SCYP log should always be made for any physical contact that is or might be perceived as, beyond what is considered appropriate, e.g. physical contact not in response to the needs of the child, for example, a team member saying to a child 'It's hug time!'
- We want to ensure unnecessary physical contact is not the norm in our programs. This could make it easier for a person with the wrong intentions to hide their behaviour.

Bathrooms and changing, e.g. dressing and undressing

Appropriate bathroom and change room arrangements ensure a safe environment for children and young people by maintaining appropriate and respectful boundaries. It also helps to ensure we are being respectful of different cultural norms.

- Make sure you are out of sight of children, young people and families when you are getting changed before or after your shift, e.g. making sure the door is closed and families can't accidentally walk in on you.
- Parents, guardians and other family members are always responsible for assisting their child or young person to change and/or visit the bathroom (e.g. if they need to change into their swimming costume during Wish Week). Starlight team members must not assist with this activity.
- If a child or young person isn't clothed on the ward (e.g. due to the dressings they require) team members should follow standard procedures of checking with the nursing staff to see if a visit is appropriate for the child or young person. If it is, follow normal procedures of working in pairs, not sitting on the child or young person's bed and not initiating any physical contact.

Uniform, Costumes, and ID

Appropriately identifying ourselves helps families and the health professionals we work with know we are part of the Starlight team.

- Starlight identification, costumes and uniforms can only be worn by a Starlight team member and must be returned once employment has ceased. The only exception is the silver star badge provided to team members in the role of Captain Starlight, which they keep as a memento of their time as Captain Starlight. This badge doesn't provide any type of external/Starlight identification.
- Starlight team members should be identifiable when interacting with families, e.g. team members should always wear their Starlight name tag or ID badge and, where appropriate, a Starlight polo, Livewire uniform or Captain Starlight costume. Examples of family interaction include at Wish Presentations and during a Get Connected shift.
- Captains' costumes can only be worn by Captain Starlight and Livewire t-shirts can only be worn by Livewire facilitators and chat hosts. These costumes and uniforms must not be worn by other Starlight team members or any other person.
- Captains should not wear their costumes journeying to and from the hospital; they need to change at the hospital. The exception is when doing Starlight Connection trips when they will get changed while collecting the Starlight vehicle and dropping it off; they don't need to change between visits to different hospitals.
- Livewire Facilitators can wear their t-shirt or hoodie but need to make sure it isn't visible until they are inside the hospital, e.g. wear a jumper or coat over it when travelling to and from work
- Captain Starlight hospital ID badges can have their Captain name on display but should also have their name (on the reverse) which can be shown if requested.
- Team members should never swap ID badges.
- Where Starlight mascot costumes, such as Starman, are loaned to a partner, it is subject to the partner following the appropriate use guidelines for the use of the costume.
- Team members must never smoke when wearing a Starlight uniform, including the Captain Starlight costume or Starlight mascot outfit.

Use, possession or supply of alcohol or drugs

Alcohol and drug use can change behaviour and lower inhibitions, which can create an unsafe environment for children and young people and increase the risk of abuse.

The prohibition of the use, possession or supply of alcohol or illegal drugs extends to where Starlight team members are likely to encounter children or young people, e.g. during an overnight stay in a remote community or a closed SER, as team members are likely to encounter children when they leave the hospital.

Transporting Children and Young People

We have clear guidelines about transporting children and young people as it can be an area of risk, particularly as it can help to facilitate grooming or one-on-one contact with a child.

- Team members must never provide personal transport to a child, young person or their family. Personal transport includes your motor vehicle, a private vehicle or a Starlight vehicle.
- Where families attend group activities, such as Wish Week and Extravaganzas, a bus or mass transportation (e.g. ferry, train) should be used to transport families to activities. Parents or guardians must remain with their child, e.g. they shouldn't be transported on separate trains.
- If families are asked by Starlight to attend an event for a media call or other fundraising activity (e.g. 5 Chefs) the family should either drive in their own car (with Starlight covering costs, such as parking) or be provided with cab vouchers. Where a team member is required to attend an event, they must not travel with the family.
- Wish transport as part of a wish is always with registered providers (e.g. limousine companies and taxis), the family car or a hire car. Where a team member is required to attend the wish, they must not travel with the family unless it is on public transport (e.g. ferry, bus).

Working with third parties

Whilst in the presence of children and young people in our programs in the hospital and online, any third party will always be accompanied by a Starlight team member.

For the Wishgranting program where third parties are engaged to visit the home e.g. a carpenter for a bedroom makeover, families will be informed about who is attending, when they will be attending and how long they are expected to be there. Parents and guardians will be asked to be at the house at all times and supervise their children. For other third-party contacts in Wishgranting e.g. hotels, tour companies, their itinerary will include all the booking details, confirmations, contact person (if needed) and contact details for Starlight.

6. Supporting Documents

- *Safeguarding Children and Young People Policy*
- *Reporting and Responding Policy*
- *Social Media Policy*
- *IT Policy*

Approvals & Revisions:

Approver Name:	Felicity McMahon
Approver Title:	Head of Programs
Approval Date:	November 2024
Effective Date:	December 2024
Review Date:	July 2026

Starlight acknowledges and pays respect to the traditional custodians of the lands on which we work, live and play.

Appendix 1: Definitions

Term	Definition
Child or young person	Starlight children and young people include:<br>• Those children and young people interact with a Starlight program. The target age range for most children and young people is four to eighteen years; however, we do reach children as young as newborns through to their twenty-second birthday.<br>• Those who have interacted with a Starlight program in the past and are still below the upper age limit of our program delivery (i.e. their twenty-second birthday).<br>• Siblings and those living with the Starlight family, e.g. foster children and cousins.<br>• Volunteers under the age of eighteen years.
Gift	A gift is anything that is given to or left for a child, young person or their family to keep by a Starlight team member. Gifts may include:<br>• Presents of any value; and<br>• Prizes.<br><br>Gifts exclude:<br>• Arts and crafts that a child may make as part of a Starlight program;<br>• Information brochures; and<br>• Activity packs are given out as part of program delivery.
Grooming	Grooming is a term used to describe what happens when a perpetrator of abuse builds a relationship with a child to abuse them at some stage. There is no set pattern concerning the grooming of children. For some perpetrators, there will be a lengthy period before the abuse begins. The child may be given special attention and what starts as a normal display of affection, such as cuddling, can develop into sexual touching or masturbation and then into more serious sexual behaviour. Other perpetrators may draw a child in and abuse them relatively quickly. Some abusers do not groom children but abuse them without forming a relationship at all. Grooming can take place in any setting where a relationship is formed, such as leisure, music, sports and religious activities, or in internet chatrooms, in social media or by other technological channels.
Starlight Team member	Board Directors, Executive team, People Managers, all paid team members and all programs volunteers, including interns (i.e. all volunteers interacting with children and young people).
SCYP Log	A written report of a breach of the Code of Conduct is completed online or by calling the SCYP Hotline on 1800 951 150 or via an online SCYP log. All reports are to be made as soon as possible and within 24 hours of behaviour or incident being observed

Starlight acknowledges and pays respect to the traditional custodians of the lands on which we work, live and play.
